# Differences in microbial community structure and nitrogen cycling in natural and drained tropical peatland soils

**DOI:** 10.1038/s41598-018-23032-y

**Published:** 2018-03-16

**Authors:** Mikk Espenberg, Marika Truu, Ülo Mander, Kuno Kasak, Hiie Nõlvak, Teele Ligi, Kristjan Oopkaup, Martin Maddison, Jaak Truu

**Affiliations:** Department of Geography, Institute of Ecology and Earth Sciences, University of Tartu, 46 Vanemuise Street, 51014 Tartu, Estonia

## Abstract

Tropical peatlands, which play a crucial role in the maintenance of different ecosystem services, are increasingly drained for agriculture, forestry, peat extraction and human settlement purposes. The present study investigated the differences between natural and drained sites of a tropical peatland in the community structure of soil bacteria and archaea and their potential to perform nitrogen transformation processes. The results indicate significant dissimilarities in the structure of soil bacterial and archaeal communities as well as *nirK*, *nirS*, *nosZ*, *nifH* and archaeal *amoA* gene-possessing microbial communities. The reduced denitrification and N_2_-fixing potential was detected in the drained tropical peatland soil. In undisturbed peatland soil, the N_2_O emission was primarily related to *nirS*-type denitrifiers and dissimilatory nitrate reduction to ammonium, while the conversion of N_2_O to N_2_ was controlled by microbes possessing *nosZ* clade I genes. The denitrifying microbial community of the drained site differed significantly from the natural site community. The main reducers of N_2_O were microbes harbouring *nosZ* clade II genes in the drained site. Additionally, the importance of DNRA process as one of the controlling mechanisms of N_2_O fluxes in the natural peatlands of the tropics revealed from the results of the study.

## Introduction

Nitrogen (N) is a naturally occurring element that is vital for all organisms, as N is component of proteins and nucleic acids. Therefore, the N cycle is one of the most important nutrient cycles in an ecosystem^1^. This cycle is driven by abiotic, decomposition, assimilative and dissimilative processes. The latter includes different microorganism-mediated pathways such as N fixation^2^, nitrification^3,4^, denitrification^5,6^, dissimilatory nitrate reduction to ammonium (DNRA)^7^ and anaerobic ammonium oxidation (ANAMMOX)^8^, as well as the newly described complete oxidation of ammonium to nitrate (comammox)^9,10^. The ongoing discovery of new processes and organisms involved in these complex systems widens our understanding of N cycling.

Tropical peatlands are particularly important in terms of nutrient cycling. Several recent studies suggest that far more peat exists in the tropics than was previously estimated^11,12^. The tropical peat area (ca 1.7 million km^2^), volume (ca. 7,268 km^3^) and carbon pool (350 Gt) may be more than three times larger from previous estimates and nearly a half of tropical peatlands can be found in South America^12^. Tropical peatlands play a crucial role in the maintenance of different ecosystem services, including sources for groundwater or surface water, biodiversity conservation, reduction of excess nutrient flows from surface waters and the sequestration and storage of atmospheric carbon^13^. Large areas of tropical peatlands (e.g., two-thirds of the Southeast Asian tropical peatlands) are drained for agriculture, forestry, peat extraction and human settlement purposes^14^. The drainage ditch network across the peatland regulates the soil water content to achieve the best conditions for different land use practices, which affect the distribution and supply of nutrients, such as carbon and N, and microbial community abundance and composition in peat^15^. Since the management of N is economically, ecologically and environmentally critical^16,17^, there is an emerging interest in understanding the ecology of microbes involved in N transformation processes in tropical ecosystems^18,19^.

The integration of soil conditions, soil microbial diversity and functional traits has proven to be an informative approach for exploring ecosystem responses to a changing environment^20–22^. Furthermore, understanding the link between changing environmental factors and microbial community dynamics provides insights into the N cycle in soil. Several studies have shown that the effect of environmental parameters on N cycling is dependent on the ecosystem type^23–25^. Although some research concerning the abundance and activity of microorganisms has been performed in tropical peatlands in Southeast Asia (reviewed by Hatano *et al*.^18^; Nurulita *et al*.^26^) and Central America^27^, studies focusing on microbial processes in South American tropical peatlands are lacking. Furthermore, modelling studies on greenhouse gas emissions, which are essential for estimations of these ecosystems’ impacts on climate change, are also limited due to the paucity of knowledge regarding microbial processes governing emissions from this region^28^.

We hypothesise that the drainage of a tropical peatland will significantly affect the soil microbial community structure and drive N transformation towards the prevalence of aerobic processes. The aim of this study was to assess the differences in the community structure of soil bacteria and archaea and their potential to perform different N transformation processes between natural and drained sites of a tropical peatland in French Guiana.

## Results

### Soil physicochemical conditions and N gas emissions from soil

**Figure 1:**
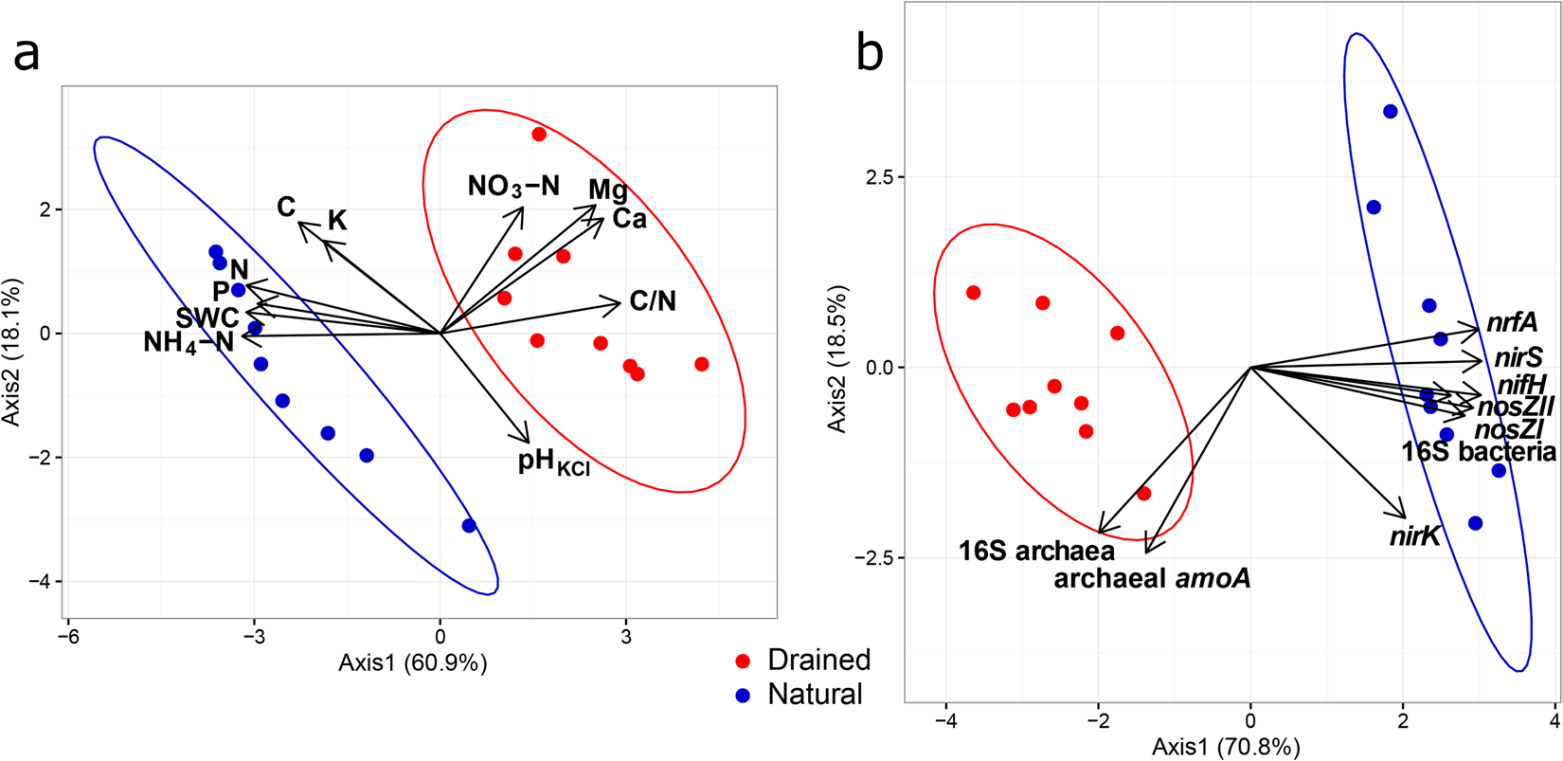
Characteristics of physicochemical and gene parameters in the natural and drained peatland sites. Principal components analysis (PCA) ordination plots with 95% confidence ellipses demonstrating the grouping of soil samples according to their physicochemical parameters (**a**) and target gene abundances (obtained by qPCR) (**b**). Abbreviations: SWC – soil water content, 16S bacteria – bacterial 16S rRNA gene, 16S archaea – archaeal 16S rRNA gene.

**Figure 2:**
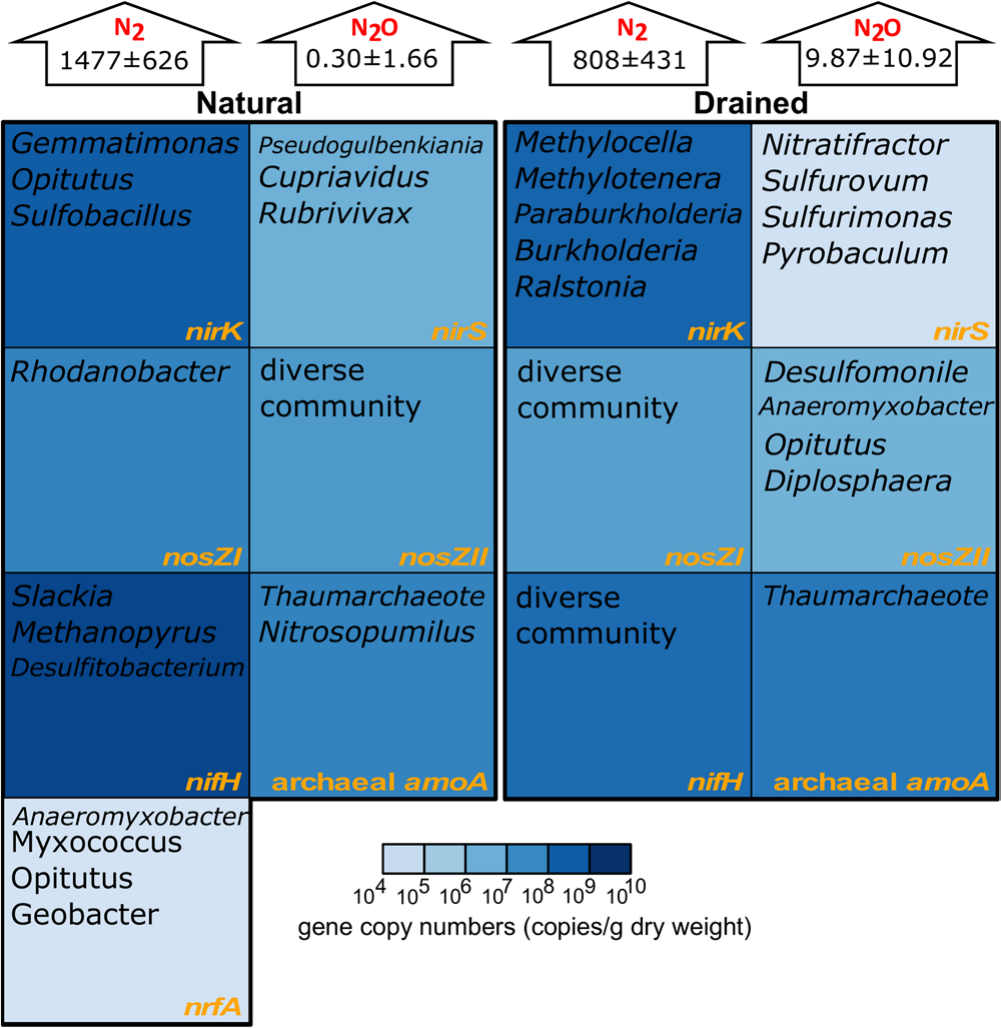
Comparison of the N-transforming microbial groups’ abundance and diversity between the top soil of natural and drained peatland sites. The most abundant microbial groups revealed by edge principal components analysis (edge PCA) (*nirK*, *nirS*, *nosZ*, *nifH* and archaeal *amoA* genes; Supplementary Fig. 1–5) and appropriately averaged phylogenetic tree (*nrfA* gene; Supplementary Fig. 6) are shown. Means and standard deviations (n = 9) of the measured nitrogen gases (N_2_ and N_2_O) emissions (μg m^−2^ h^−1^) are shown.

The physicochemical conditions in the top 10-cm layer of soil were significantly different between the natural and drained site (Fig. 1a and Supplementary Table 1). The natural and drained sites differed in their N_2_O and N_2_ emission from soil (Fig. 2). The average N_2_O flux from the natural site was significantly lower (F = 6.98, p < 0.05) than that for the drained site. The N_2_ emission potential was highly variable in the 0- to 10-cm soil layers of both study sites, but it was significantly greater at the natural site (F = 6.76, p < 0.05).

### Soil microbial community abundance and phylogenetic composition

Quantitative PCR (qPCR) results showed that the proportion of bacteria and archaea in prokaryotic communities differed between the natural and drained soils (F = 83.51, p < 0.001, Supplementary Table 2). The archaeal abundance exceeded the bacterial abundance by more than one order of magnitude in the drained site, whereas at the natural site, the two were almost equally represented in the community. The total bacterial abundance was higher (F = 38.89, p < 0.001) and the archaeal abundance lower (F = 17.08, p < 0.01) at the natural site than at the drained site (Fig. 1b).

**Figure 3:**
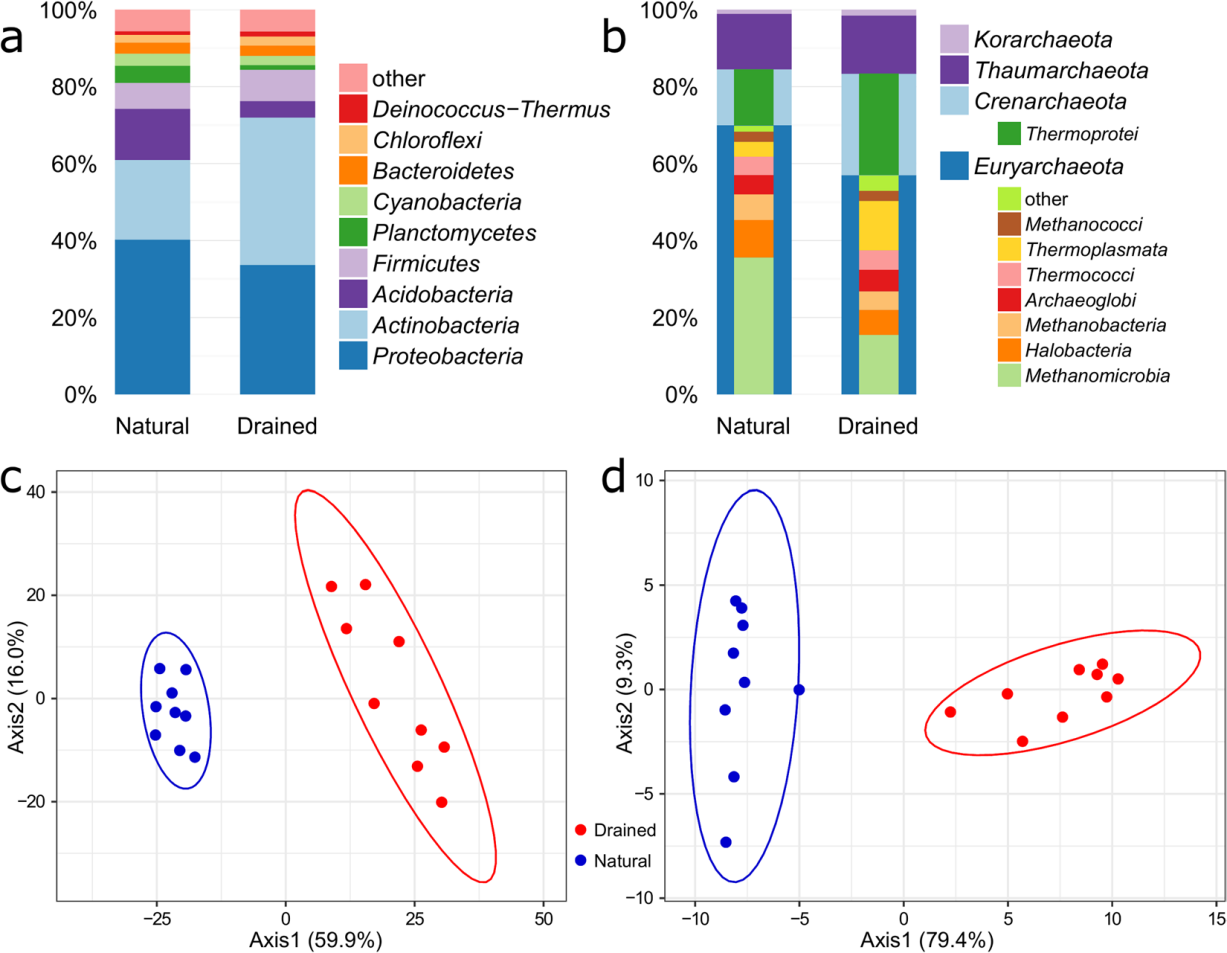
Microbial community structures of the natural and drained peatland sites from metagenome. The dominant bacterial phyla (**a**) and archaeal phyla (column edges) and classes (column interior) (**b**) in soil samples. PCA ordination plots with 95% confidence ellipses demonstrating the grouping of soil samples according to the proportions of bacterial (**c**) and archaeal (**d**) genera.

The structure of the bacterial and archaeal communities also differed between the natural and drained sites revealed from the analyses of metagenomes (Fig. 3). Overall, 32 different bacterial phyla were identified. The average proportions of nine dominant bacterial phyla were different by 0.1 to 17.5% between the sites (Fig. 3a) and statistical analysis confirmed significant difference (F = 32.46–760.22, p < 0.001 in all cases) between almost all of these phyla (except for *Proteobacteria*). The natural and drained sites also significantly differed in their bacterial genera composition (F = 53.86, p < 0.001; Fig. 3c, Supplementary Fig. 7), although *Mycobacterium*, *Conexibacter*, *Burkholderia*, *Rhodoplanes*, *Pseudomonas* and *Paenibacillus* were the dominant genera at both sites.

The dominant archaeal phyla were *Euryarchaeota*, *Crenarchaeota* (class *Thermoprotei*), *Thaumarchaeota* and *Korarchaeota* at both study sites (Fig. 3b), although three of them were significantly different between sites (F = 11.75–216.26, p < 0.01 for *Korarchaeota* and p < 0.001 for *Euryarchaeota* and *Crenarchaeota*). At the natural site, more than half of the *Euryarchaeota* belonged to the class *Methanomicrobia*, whereas *Methanomicrobia* and *Thermoplasmata* were the most abundant euryarchaeal classes at the drained site. PCA of the archaeal genera showed that two distinct clusters were formed for both study sites (F = 134.70, p < 0.001; Fig. 3d). “*Ca*. Nitrosotenuis”, *Nitrosopumilus*, *Thermococcus*, *Methanobacterium*, “*Ca*. Nitrosopelagicus”, *Pyrococcus*, *Methanobrevibacter*, *Methanocaldococcus*, *Geoglobus*, *Methanothermobacter*, *Ferroglobus* and *Halobacterium* were among the most abundant genera at both study sites. A number of archaeal genera were differentially abundant between the natural and drained sites (Supplementary Fig. 8).

Combining the proportions obtained from metagenomes with 16S rRNA gene abundances (qPCR data), the obtained abundances of all dominant bacterial phyla were higher at the natural site and archaeal phyla at the drained site (Supplementary Fig. 9).

### Abundance and diversity of N-transforming microbial groups in the study soils

*nirS*, *nirK*, *nosZI*, *nosZII*, *nifH* and archaeal *amoA* genes were detected in all studied soil samples, whereas *nrfA* genes were detected only in soil samples from the natural site (Figs 1b and 2, Supplementary Table 2). Bacterial *amoA* and ANAMMOX-specific 16S rRNA genes were not detected from either of the sites. These results were also confirmed by metagenomic analysis, in which neither *hzsA* (ANAMMOX) nor bacterial *amoA*/*pmoA* genes were detected in the study site samples, and *nrfA* genes were not detected in samples from the drained site.

A clear separation between the natural and drained sites was revealed by PCA according to the abundance of functional genes (Fig. 1b). The abundances of *nirS*, *nosZI*, *nosZII* and *nifH* were significantly higher in the natural site soil (F = 57.09–521.85, p < 0.001 for *nirS*, *nosZI* and *nifH*; F = 23.68, p < 0.01 for *nosZII*), and the archaeal *amoA* abundance was higher in the drained soil (F = 7.34, p < 0.05).

The proportions of *nirS*, *nirK*, *nosZI*, *nosZII* and *nifH* in prokaryotic communities were significantly higher in the natural site soil than in the drained site soil (F = 70.35–460.54, p < 0.001 in all cases), whereas the proportions of archaeal *amoA* appeared to be fairly similar at the natural and drained sites (Supplementary Table 2).

The levels of *nirS/nirK* and *nosZ*/*nir* were significantly higher (F = 72.56–104.05, p < 0.001 in both cases) at the natural sites than at the drained sites (Supplementary Table 2). The ratio of *nosZI*/*nosZII* ranged from 1.2 to 35.9 across the study sites, and this parameter was not significantly different between the two sites.

Metagenomic analysis revealed the presence of a comammox bacterium (“*Ca*. Nitrospira inopinata”) whose sequence abundance was 0.14–0.19% and 0.09–0.13% among all classified bacteria at the natural and drained sites, respectively. However, quantification of this group failed when using the available comammox *Nitrospira* specific primers due to unspecific amplification.

**Figure 4:**
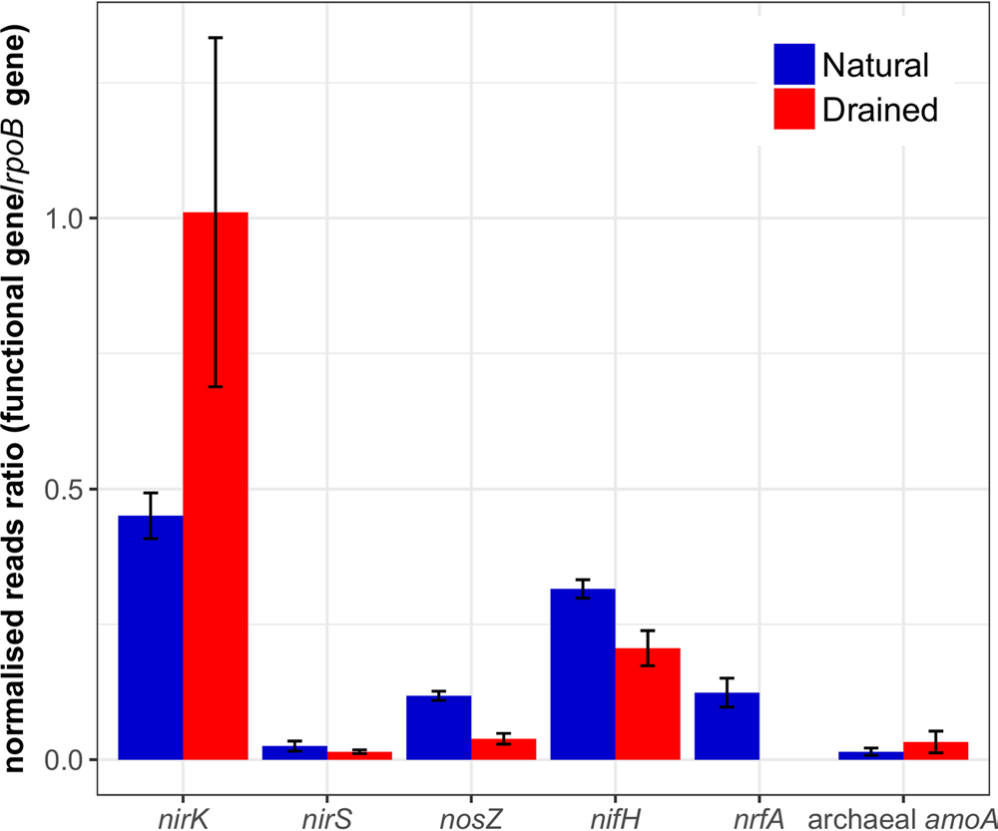
Average values (n = 9) and standard deviations of proportions of studied nitrogen cycling gene reads in the metagenome dataset for natural and drained peatland sites. Functional gene read abundances are shown as proportions of total *rpoB* (RNA polymerase) gene read abundance. Read counts were normalised according to gene length.

The proportion of studied nitrogen cycling genes reads in the metagenome dataset for natural and drained peatland sites were shown in Fig. 4. Based on the balanced weighted phylogenetic diversity (BWPD) index, we found that *nirK* (t = 5.55, p < 0.001), *nirS* (t = –3.25, p < 0.01), *nosZ* (t = 2.99, p < 0.01), *nifH* (t = 6.73, p < 0.001) and archaeal *amoA* (t = –6.37, p < 0.001) gene phylogenetic diversities were significantly different at the natural and drained sites (Supplementary Table 3). *nosZ* alpha diversity and potential of N_2_ emission were significantly positively correlated (R = 0.78, p < 0.05) at the natural site but *nirS* alpha diversity and potential of N_2_ emission were significantly negatively related (R = –0.82, p < 0.01) at the drained site.

**Figure 5:**
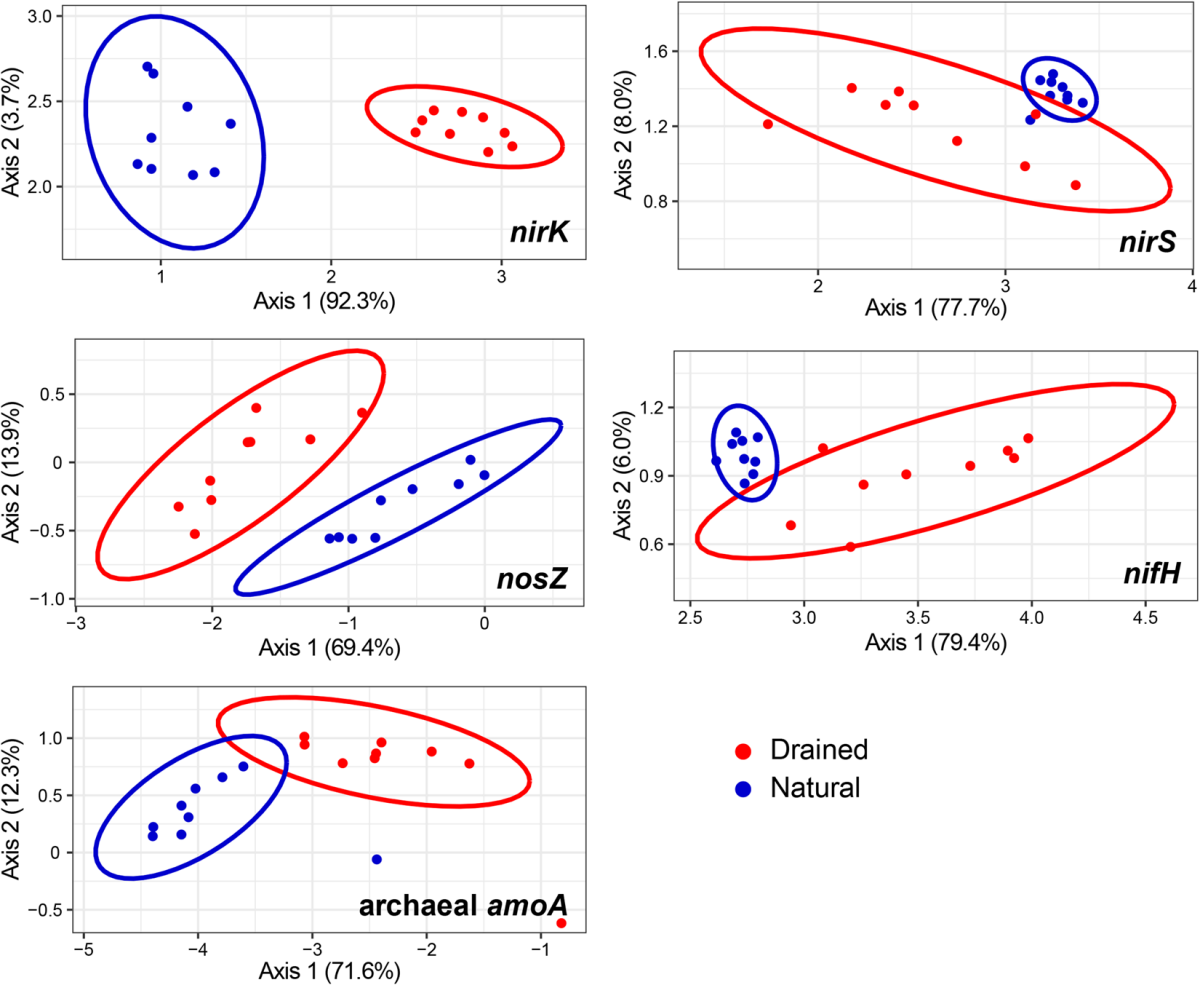
Ordination of soil samples for different N-transforming microbial groups in the natural and drained peatland sites. Edge PCA ordination plots with 95% confidence ellipses demonstrating the grouping of soil samples according to the diversity of *nirK* (Supplementary Fig. 1), *nirS* (Supplementary Fig. 2), *nosZ* (clade I and II) (Supplementary Fig. 3), *nifH* (Supplementary Fig. 4) and archaeal *amoA* (Supplementary Fig. 5) gene-possessing microbes.

Edge PCA results indicate to variable extent grouping of soil samples according to sampling location for *nirK*, *nirS*, *nosZ* (clade I and II), *nifH* and archaeal *amoA* gene phylogenetic diversity (Figs 2 and 5). PERMANOVA (permutational multivariate analysis of variance) confirmed the significance of the differences in N-cycling microbial community structure between the natural and drained sites (F = 17.80–203.0, p < 0.001 in all cases). In all cases, the first principal component from the edge PCAs provided main separation of the samples.

For *nirK*-harbouring microorganisms, the edge PCA first principal component was related to the higher relative abundance of the genera *Gemmatimonas*, *Opitutus* and *Sulfobacillus* at the natural site, while the genera *Methylocella*, *Methylotenera*, *Paraburkholderia*, *Burkholderia* and *Ralstonia* were more abundant at the drained site (Supplementary Fig. 1). The diversity of *nirS*-harbouring microorganisms varied along the first PCA axis for the drained site. The difference in *nirS*-harbouring microbes between the natural and drained soils was primarily due to the higher contribution of the genera *Pseudogulbenkiania*, *Cupriavidus* and *Rubrivivax* at the natural site and of *Nitratifractor*, *Sulfurovum*, *Sulfurimonas* and *Pyrobaculum* at the drained site (Supplementary Fig. 2). A superposition of the *nosZI*-harbouring microbial community composition on the phylogenetic tree showed that the drained site has a diverse and heterogeneous community, while only the *nosZI* gene-possessing genus *Rhodanobacter* was abundant at the natural site (Supplementary Fig. 3). In contrast, the natural site possessed a diverse community of *nosZII*-harbouring microbes, while the drained site was colonised with only four genera: *Desulfomonile*, *Anaeromyxobacter*, *Opitutus* and *Diplosphaera*. The edge PCA of *nifH*-harbouring microorganisms indicated that the natural site had a higher abundance of *Slackia*, *Methanopyrus* and *Desulfitobacterium*, while the drained soil had a more diverse set of genera (Supplementary Fig. 4). The separation of *amoA*-harbouring archaea between the natural and drained sites occurred primarily along the edge PCA first principal component and was related to the higher abundance of *Nitrosopumilus* at the natural site; other differences were attributed to an uncultured thaumarchaeote (Supplementary Fig. 5). *nrfA* gene-possessing microbes were detected only at the natural site (mainly from the genera *Anaeromyxobacter*, *Myxococcus*, *Opitutus* and *Geobacter*) (Supplementary Fig. 6).

Similar patterns for community differences across the sites were observed for *nirK*, *nirS*, *nosZ*, *nifH* and archaeal *amoA* functional genes, as indicated by the Procrustes analysis, with highly significant goodness-of-fit measures (M^2^ ranging from 0.31 to 0.55; p < 0.001 in all cases, except between *nirS* and archaeal *amoA* genes (p < 0.01)) (Supplementary Table 4). Looking at the results of the beta diversity partitioning for N-transforming microbial groups (Supplementary Table 3), it appeared that in case of *nirK* gene-possessing microbes the replacement was dominating process, which accounted for 89.5% of the total beta diversity. In case of other studied N-cycle genes the replacement component contribution was still high but the richness difference accounted for one third or more of the total beta diversity.

### Relationships between target genes, physicochemical parameters and gas emissions

**Figure 6:**
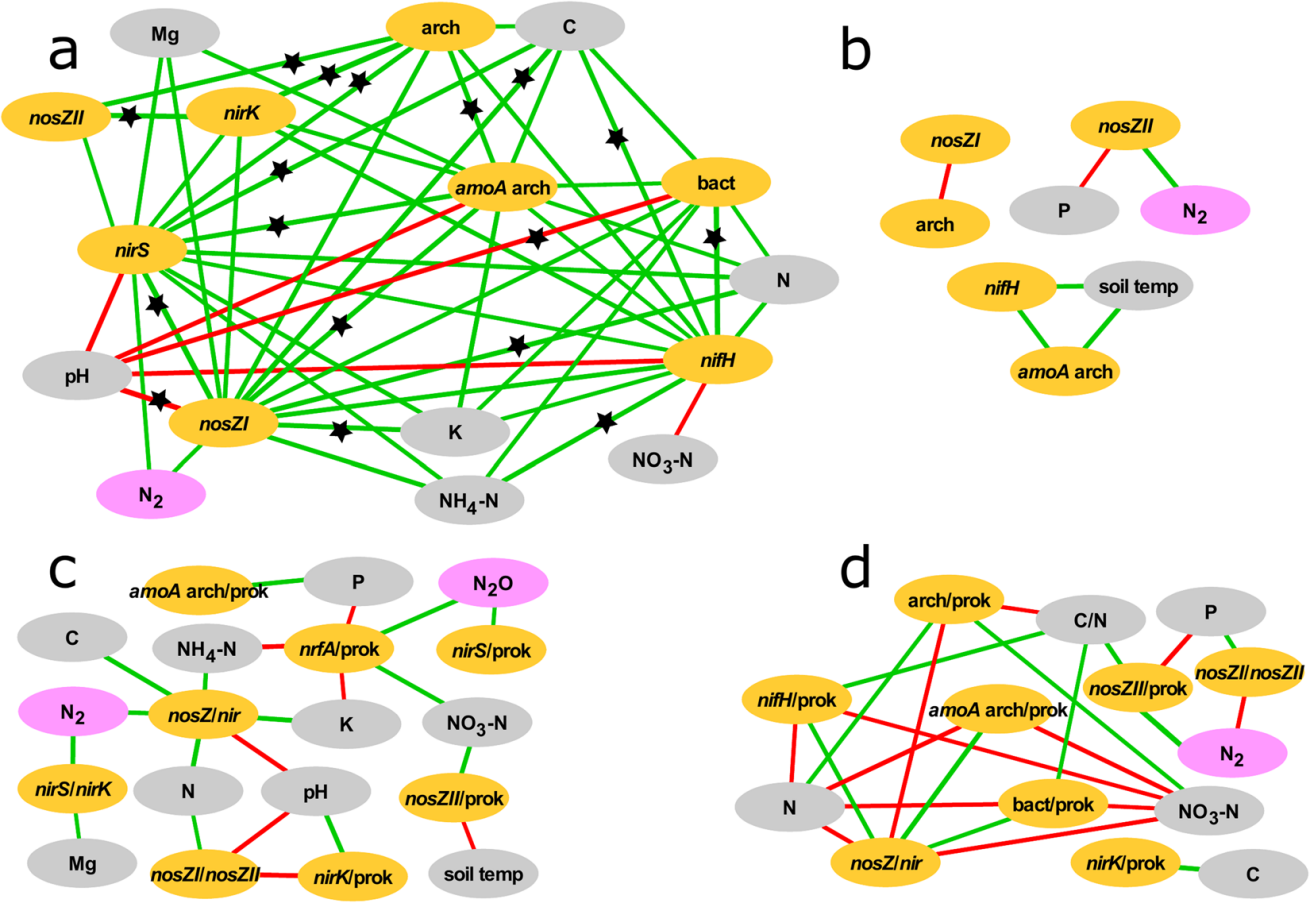
Spearman correlation networks (p < 0.05) for target gene abundances (obtained by qPCR) (a – natural site, b – drained site) and target gene proportions and ratios (obtained by qPCR) (c – natural site, d – drained site). The pair wise Spearman correlation was calculated for each target gene parameter and environmental factor pair (exact R and p values are shown in Supplementary Tables 5 and 6). The Benjamini-Hochberg corrected (p < 0.05) Spearman correlations are specified with black stars. Green is used to represent positive relationships, and red indicates negative relationships; yellow stands for gene abundances, grey for soil physicochemical variables and pink for gaseous parameters. Abbreviations: soil temp – soil temperature, bact – bacterial 16S rRNA gene abundance, arch – archaeal 16S rRNA gene abundance, prok – prokaryotic community (total 16S rRNA gene proportion), N_2_ – potential N_2_ emission, N_2_O – N_2_O emission rate.

The data analysis detected several statistically significant relationships between target gene parameters and environmental factors, but the patterns of these relationships were different for the drained and natural sites (Fig. 6, Supplementary Tables 5 and 6). At the natural site, the archaeal 16S rRNA gene abundance was strongly related to most of the detected functional gene abundances, while only two significant correlations were found between gene abundances in the drained soil. The relationships between bacterial 16S rRNA gene abundance and *nosZI* and archaeal *amoA* and the particularly strong relationship with *nifH* abundance were revealed by a correlation analysis of the natural site. For the drained site, strong correlations between the bacterial and archaeal 16S rRNA proportions and the *nifH* and archaeal *amoA* proportions were detected. The *nifH* abundance was found to be related to most of the studied denitrification pathway genes at the natural site, while at the drained site, the proportion of *nifH* was related to *nosZI* and archaeal *amoA* proportion in the prokaryotic community. We did not detect any relationships between *nrfA* and other targeted genes for either of the study sites.

The soil C, N and C/N content was related to most of the gene parameters, but the effect differed between the two study sites. In addition, the pH had a strong effect on many gene parameters in the natural site soil, but this was not the case for the drained site. The *nrfA* proportion in the natural site community showed correlations with the soil chemical composition, while no correlations were found between this gene and environmental parameters in the drained soils.

Abundances and ratios of different denitrification genes were significantly related to the potential of N_2_ emission from the topsoil layer. The proportion of *nirS* and *nrfA* in the natural site microbial community correlated with N_2_O emission from the soil.

Distance-based regression analysis showed that soil chemical variables (especially NO_3_-N) explained significant amount of the variation in the functional gene-based microbial community structure (Table 1).

**Table 1:** Results of a distance-based regression analysis showing soil physicochemical variables explaining the variation in target gene community structure (p < 0.05).

Target gene	Physicochemical parameter	Variation explained (%)
*nirK*	Ca, NH_4_-N, NO_3_-N, K	68.0
*nrfA*	pH	44.5
*nifH*	NO_3_-N, Mg	32.6
*nosZ*	Ca, NO_3_-N, C, K	26.4
*nirS*	NO_3_-N, Mg	26.1
archaeal *amoA*	Ca, NO_3_-N	12.0

## Discussion

In this study, the effect of drainage on the phylogenetic composition of a microbial community and its genetic N transformation potential was linked for the first time to the site-specific characteristics of a tropical peatland. We applied a combined methodological approach by utilizing advantages of both methods (e.g., the broad-spectrum profile provided by metagenomics combined with higher sensitivity and quantitative capacity of qPCR). This approach could compensate for the weaknesses such as sequencing depth issues and adequacy of reference databases for metagenomic analysis^29^ or low-throughput, and limited availability and coverage of primers and nonspecific amplification for qPCR analysis^30^.

Our results show that the structure of a soil microbial community is significantly different in natural and drained sites of this ecosystem. At the natural site, the archaeal proportion was almost equal to the bacterial proportion in the prokaryotic community, while at the drained site, the proportion of archaea was notably higher.

Similar to most of the natural soils^22,31^, *Proteobacteria*, *Actinobacteria*, *Acidobacteria* and *Firmicutes* were among the most dominant bacterial phyla at both study sites, but the proportions of the most abundant phyla were not similar for the natural and drained sites.

Our results agree with a meta-analysis study, which found similar differences in bacterial phyla proportions (except Planctomycetes, whose proportion was increased) in tropical soils before and after the conversion of natural sites into agricultural systems^31^. Found at the natural site, the members of *Acidobacteria*, which have a broad range of transporters for the uptake of different substrates, have an advantage in complex environments and in adaptation to oligotrophic conditions^32^, and *Proteobacteria*, which are known to be important in the C, S and N cycles^33^, were more abundant. At the drained site, the proportion of *Actinobacteria* and *Firmicutes*, which are involved in the degradation and mineralisation of plant and humic materials in soil^34–36^, was higher compared to the natural site.

Our results highlight the importance of archaea, especially in a drained tropical peatland. Differences in the proportions of *Euryarchaeota* and *Crenarchaeota* between the natural and drained site were significant. The proportion of organic matter mineralising archaea (euryarchaeal classes *Thermoplasmata*, *Archaeoglobi* and *Thermococci*, as well as the phyla *Crenarchaeota* and *Korarchaeota*^37^) was approximately 20% higher in the drained soil, at the expense of the methanogenic euryarchaeal class *Methanomicrobia*. In addition, a difference between organisms performing the same methanogenic function (smaller proportion of *Methanomicrobia* and bigger proportion of *Thermoplasmata*) was detected at the drained site. Similar trends in the proportions of *Methanomicrobia* and *Thermoplasmata* were reported in an upper layer of peat (0–30 cm) in pristine and boreal ecosystems (i.e., bog, fen, spruce swamp forest) after long-term drainage^38^. The results of this study demonstrate that the proportion of the phylum *Crenarchaeota* involved in the S cycle (e.g., *Caldivirga* in sulphidogenesis and *Sulfolobus* and *Metallosphaera* in sulphide and S oxidation^37^) was twice as high in the drained peat compared to the natural peat.

Although the soil carbon content was about 5% lower compared to the natural, it was still high (around 30%) at the drained site. On the other hand, the organic matter quality was different from the natural site (indicated by C/N ratio) and more suitable for the archaeal metabolic activity at the drained site (confirmed by the significant negative relationship between proportion of archaea in the microbial community and C/N in soil). The lower ammonia content and higher nitrate concentration refer to the higher nitrification activity that is conducted by autotrophic nitrifying archaea whose proportion was also higher in the prokaryotic community of the drained site. One factor that can be related to the differences in archaeal (including ammonium-oxidising archaea (AOA)) abundances between the studied sites can be the more pronounced effect of physical factors such as temperature in upper layer of drained soils. Almost 2 °C higher temperature was recorded at the sampling time in the drained peat. Studies have shown that bacteria and archaea expressing similar activity can have different temperature preferences. Wu *et al*.^39^ showed that AOA have higher temperature optimum than ammonium-oxidising bacteria (AOB) and proved autotrophic growth of AOA under warmer experimental conditions.

We did not detect bacterial *amoA* in the studied tropical peatlands with the applied tools, while archaeal *amoA* genes were abundant at both study sites. These results are in agreement with Lu *et al*.^40^, who found a similar trend in acidic tea orchard and forest soils (pH 3.8–5.4). Because of the highly efficient anabolic pathways of AOA, which provide an ecological advantage compared to AOB in some environments^41^, AOA are known to be a dominant ammonia oxidiser in a wide range of soils^19,42,43^. Archaeal *amoA* gene diversity was substantially higher in the drained peatland soil and both replacement and to lesser extent richness difference contributed to dissimilarity in archaeal *amoA* communities between two study sites. We found higher abundance and proportion of different *amoA*-harbouring *Thaumarchaeota* members and lower proportion of *Nitrosopumilus* in the drained site soil. The study results also indicate that the soil chemical composition (including pH and C and N concentration) was strongly related to the abundance of AOA at the natural site. Soil pH has been shown to be a key factor for controlling the abundance and community composition of AOA, but many other soil characteristics (e.g., salinity, temperature, water, N, organic C and O content) may also affect the AOA community structure^42,43^.

Although the presence of a recently discovered complete nitrification (comammox) process has been already shown in several different ecosystems^9,10,44^, its ecological background remains unclear. In our study, the results from a metagenomic analysis suggest that this microbial group may be present in both tropical peatland soils.

Denitrification is shown to be a major microbial pathway for nitrate reduction and losses from soil in natural environments^45,46^. We focused on the key functional genes involved in the modular denitrification pathway, where either the *nirS* or *nirK* gene encodes nitrite reductase and the *nosZ* clade I or II gene encodes nitrous oxide reductase. Not all denitrifiers have a complete denitrification pathway^47^, and molecular studies have shown that the gene copy abundance of *nir* usually significantly exceeds that of *nosZ* in various environments^24,48^. We also found by applying qPCR that the balance between *nosZ* and *nir* genes was in favour of the latter genes at both study sites. In our study, the abundance and proportion of organisms harbouring the targeted denitrification genes (*nirS*, *nosZI* and *nosZII*) were higher in the drained soils; however, *nirK*-type denitrifiers were equally represented at both study locations. The abundance of *nirS*-type and *nosZI*-type denitrifiers showed a similar pattern in response to the edaphic factors in the natural soil. Our results are consistent with those of Stone *et al*.^49^, who found that the abundances of *nirS* and *nosZ* were positively correlated with soil C, N and P concentrations in humid tropical forests in Puerto Rico. The observed correlations suggest that *nirS*-type and *nosZI*-type denitrifiers play an important role in controlling N_2_O and N_2_ gas fluxes in the natural peatland soil, while microbes harbouring the *nosZII* gene more likely perform N_2_O transformation to N_2_ at the drained site. It could be assumed that the discrepancy between the denitrification communities of the two sites might be caused by a selective pressure of different environments in which two different N_2_O reductase mechanisms are preferred^48^. We cannot exclude that the obtained differences in N cycling genes’ abundances between the study sites could be partly related to the selected primers that could be more specific for members of the N-cycling microbial community at one of the study sites^30,50^.

The metagenomic data indicate that the community structure of organisms possessing either *nir* genes differed between the study sites. The *nirK* gene diversity was higher at the natural site and the dissimilarity between *nirK* possessing communities two locations was dominated by replacement process. The *nirK*-type denitrifiers belonging to the genera *Gemmatimonas*, *Opitutus* and *Sulfobacillus* were dominant in the natural site soil, while the genera *Methylocella*, *Methylotenera*, *Paraburkholderia*, *Burkholderia* and *Ralstonia* were predominant at the drained site. The *nirS*-type denitrifiers belonging primarily to *Pseudogulbenkiania*, *Cupriavidus* and *Rubrivivax* were observed at the natural site, while at the drained site their diversity was slightly higher. *Nitratifractor*, *Sulfurovum*, *Sulfurimonas* and *Pyrobaculum nirS*-type denitrifiers were dominant at the drained site. According to Graf *et al*.^51^, the majority of the abovementioned *nirS*-type denitrifiers also possess the *nosZ* gene, while only half of the listed *nirK*-type denitrifiers have the ability to reduce N_2_O. At the natural site, in addition to the diverse community of *nosZII*-harbouring microbes, *nosZI* gene-possessing genus *Rhodanobacter* was found to be abundant. The genus *Rhodanobacter* has been shown to be an important group of denitrifiers in acidic soils^52^. At the drained site, a diverse community of *nosZI*-harbouring organisms and *nosZII*-type denitrifiers (*Desulfomonile*, *Anaeromyxobacter*, *Opitutus* and *Diplosphaera*) were predominant. The results indicate that the soil C and N content significantly affects the denitrifying community structure in tropical peatland soil. In addition, denitrification ability has been described for several archaea including the genera observed in the studied tropical peatland (*Haloferax*, *Halobacterium* and *Ferroglobus*)^53^.

Another N reduction pathway, DNRA, is favoured in competition with denitrifiers under nitrate-limited conditions with suitable organic C source availability^54^. Depending on the environmental conditions, microbes performing DNRA may release N_2_O as a by-product of the reduction process or may reduce N_2_O produced by themselves or other microorganisms^55^. We detected *nrfA*-specific sequences (mainly from the genera *Anaeromyxobacter* and *Myxococcus)* at only the natural site. The importance of DNRA as one of the controlling mechanisms of N_2_O fluxes in the natural peatlands of the tropics can be assumed from the positive relationship between the *nrfA* proportion and N_2_O emission values. Templer *et al*.^56^ reported that DNRA rates were much higher than N_2_O production rates from denitrification (approximately 35% of gross nitrification) in a humid tropical forest soil in Puerto Rico.

In addition to DNRA, which is generally considered a process that conserves N in the ecosystem even though many microorganisms conducting DNRA also produce N_2_O^54,55^, biological N fixation (symbiotic and free-living) is another process that promotes N retention in soil^25^. Current evidence suggests that terrestrial ecosystems receive a critical N input from free-living diazotrophs, especially when the system includes a limited number of symbiotic N_2_-fixing plants (roots infected by *Rhizobia*, *Bradyrhizobia* or actinomycetes)^57^. In this study, free-living N_2_-fixing microbes were responsible for the differences in N-fixing microbial communities between the natural and drained sites. The soil nitrate was primary factor affecting this community structure. The metagenomic data indicate that the N_2_-fixer diversity was higher in the drained soil than in the natural soil, whereas the abundance and proportion of N_2_-fixing organisms (obtained by qPCR) was lower. Like all soil microorganisms, N_2_-fixers are affected by a wide variety of abiotic and biotic factors in different ecosystems (forests, grasslands, oceans, etc.) worldwide (reviewed by Reed *et al*.^57^). The results of the present study showed that only soil temperature is correlated with the abundance of N_2_-fixing organisms in a drained tropical peatland, while a number of different chemical parameters regulated the abundance of N_2_-fixers in a wet natural site. In addition to bacteria, N_2_ fixation is shown to be widespread among methanogenic *Euryarchaeota*^53^, which were significantly more abundant at the natural site.

Hu *et al*.^58^ obtained an enriched ANAMMOX culture from a N-loaded peat soil and showed some potential for ANAMMOX activity in the top 10-cm layer of peat. Nevertheless, neither metagenomic nor qPCR analyses detected ANAMMOX organisms from the studied tropical peatland sites, confirming that the ANAMMOX process plays only a minor role in most terrestrial soils^59^.

Our results indicate the coupling of several N transformation processes in the studied tropical peatland soil and a strong effect of soil drainage on the microorganisms performing these transformations. Nelson *et al*.^60^ suggested that the abundant soil microbial groups utilising a given N pathway usually support prokaryotes that can use other N pathways, and many microorganisms are able to perform multiple functions in the N cycle. For example, some microorganisms conducting DNRA are capable of N_2_O reduction to N_2_ because they possess the *nosZ* gene (mainly clade II)^61^. This study also demonstrated that the proportions of *nrfA* and *nosZ* clade II genes were positively correlated at the natural site. Additionally, numerous other positive relationships were revealed by the juxtaposition of the different microbial community abundances or proportions at the study sites, although the causes of these relationships are not yet entirely clear.

Analysis of N-cycle genes phylogenetic diversity in the current study is based on bioinformatics pipeline with the multiple steps. The results of each intermediate data analysis step have an inherent uncertainty and potentially impact the as-yet-unmeasured statistical significance of downstream analyses. Some of the studied N-cycle genes have highly conserved motifs separated by highly variable regions, and the short sequence limit of metagenome sequences might be insufficient for detecting a real hit from distant homologs that do not share the same function. We encountered this type of the problem of possible false positives with *nirS* and especially in case *nirK* gene (Supplementary Figs 1 and 2). In order to overcome this problem, improved bioinformatic data analysis methods are needed to reliably detect and quantify target genes in short-read metagenomes.

The results of this study show that modifications in the water regime of tropical peatlands may cause substantial shifts in the microbially mediated N cycle but more extensive studies are needed to confirm the revealed tendencies.

## Methods

### Site description, soil and gas sampling and analyses

The studied peatland is located in the northern region of French Guiana, where the average monthly temperature is approximately 26 °C, with little variation between seasons, and the average annual rainfall is 3000–4000 mm^62^. *In situ* measurements and soil sampling were performed at two sites of this peatland in October 2013 (dry season): a natural site located close to the village of Tonate (4°59'27“N, 52°27'14“W) and a drained site near the town of Kourou (5°09′42″N, 52°39′06″W). Further detailed information regarding the sampling sites, soil and gas sampling as well as analyses is provided in the Supplementary Information.

### DNA extraction

Three parallel DNA extractions were performed from each sample (0.15 g of soil) with a PowerSoil DNA Isolation kit (MO BIO Laboratories Inc., Carlsbad, CA, USA) according to the manufacturer’s instructions. Homogenisation of samples was performed using a Precellys® 24 (Bertin Technologies, Montigny-le-Bretonneux, France) at 5000 rpm for 20 s. The Infinite M200 spectrophotometer (Tecan AG, Grödig, Austria) was used to determine quality and quantity of extracted DNA (Supplementary Table 7). The parallel extracts were pooled and stored at −20 °C.

### Preparation of DNA libraries, sequencing and data processing

DNA samples were purified using a Genomic DNA Clean and Concentrator kit (Zymo Research, Irvine, CA, USA) according to the protocol provided by the manufacturer. Paired-end sequencing libraries were constructed for each sample using the TruSeq DNA PCR-Free Library Preparation kit (Illumina, San Diego, CA, USA) according to the manufacturer’s instructions. DNA concentrations of individual samples were quantified with a Qubit Fluorometer (Thermo Fisher Scientific Inc., MA, USA), and finally, the sample DNA was pooled in equal proportions. The library was sequenced using the NextSeq. 500 Illumina sequencing system (Illumina, San Diego, CA, USA). See the Supplementary Information and Supplementary Table 7 for further analysis details.

### Quantitative PCR

qPCR was applied to evaluate the bacterial and archaeal community abundance by quantifying the abundances of specific 16S rRNA genes and to evaluate the genetic potential of the following N transformation processes by quantifying the respective functional genes: denitrification (*nirS*, *nirK*, *nosZ* clade I and *nosZ* clade II), N fixation (*nifH*), DNRA (*nrfA*), nitrification (bacterial and archaeal *amoA*), comammox (*amoA* clade A and clade B) and ANAMMOX (ANAMMOX-specific 16S rRNA genes). See the Supplementary Information and Supplementary Tables 8 and 9 for further details.

### Statistical analyses

In all tests, statistical significance was determined at a 95% confidence level. For data analyses, samples were grouped according to the study sites: natural (n = 9) and drained (n = 9) soil groups. Separate PCAs were performed on soil physicochemical and microbiological data (gene copy numbers, proportions of bacterial and archaeal genera) using the R package ade4 v. 1.7–4^63^. To evaluate the significance of the differences between study sites with respect to physicochemical variables, gene parameters, phylogenetic data (the log-ratio (clr) transformation^64^ and genetic marker *rpoB* gene was used for the normalisation of metagenomic reads) and emission values, multivariate linear models were constructed, and models were tested using the anova function in R package mvabund v. 3.11.9^65^. Spearman’s rank correlation coefficients were used to assess the relationships between environmental factors and target gene abundances as well as the relationships between different gene abundances. The p-values were also adjusted for the false discovery rate by the Benjamini-Hochberg method with significance at p < 0.05. Spearman correlation networks were visualised with Cytoscape v. 3.4.0^66^.

In case of each N-cycling gene, edge PCA was performed to detect differences between soil samples containing closely related taxa^67^, and graphics were produced with the R package ggplot2 v. 2.1.0^68^ and the Archaeopteryx tree viewer v. 0.9920^69^. *nrfA* phylogenetic tree with edges fattened in proportion to the number of reads was made for natural site by guppy tool from pplacer suite^70^. BWPD indices, which account for abundance and are robust to sampling depth differences between samples, were computed at theta 0.5 for each N-cycling gene to investigate the diversity of the soil community at the natural and drained sites (alpha diversity)^71^. Alpha diversity indices were compared between the natural and drained sites using t-test. The Spearman correlations were computed between alpha diversities and N gas emissions. To evaluate beta diversity among sites, we calculated percentage difference indices and decomposed beta diversity into its components of replacement and richness difference for each N-cycling gene using the R package adespatial v. 0.0-9^72^.

For PCA and edge PCA, differences in microbial community structure between the natural and drained sites were evaluated using PERMANOVA with 9999 permutations, using the R package vegan v. 2.4-1^73^. The same package was used to assess pairwise marker gene community structure concordance, and the ordination results of different edge PCAs were compared with a Procrustes rotation using the protest function with 9999 permutations^74^.

Distance-based regression analysis was applied using the DISTLM program^75^ with the forward selection procedure and 9999 permutations to identify soil physicochemical variables explaining variations in marker gene community structure.

Beta diversity evaluation, PERMANOVA and distance-based regression analysis based on Kantorovich-Rubinstein distance matrices, which were calculated by guppy tool from pplacer suite^70^.

### Data availability

All raw sequencing reads have been deposited in the European Nucleotide Archive under the study accession number PRJEB21930. The data that support the findings of this study can be requested from M.E.

## Electronic supplementary material

Supplementary_information
