# Supplementary material for: Differences in microbial community structure and nitrogen cycling in natural and drained tropical peatland soils
Source: Sci Rep. 2018 Mar 16;8:4742. doi: 10.1038/s41598-018-23032-y (PMC5856767; doi:10.1038/s41598-018-23032-y)
Supplement: Supplementary file 1 — Supplementary_information [file 41598_2018_23032_MOESM1_ESM.pdf]

SUPPLEMENTARY INFORMATION

Differences in microbial community structure and nitrogen cycling in natural and drained tropical peatland soils

Mikk Espenberg[1*], Marika Truu[1], Ülo Mander[1], Kuno Kasak[1], Hiie Nõlvak[1], Teele Ligi[1], Kristjan Oopkaup[1], Martin Maddison[1] & Jaak Truu[1]

[1]Department of Geography, Institute of Ecology and Earth Sciences, University of Tartu, 46 Vanemuise Street, 51014 Tartu, Estonia

*Corresponding author: mikk.espenberg@ut.ee

SUPPLEMENTARY METHODS

Site description and soil sampling

The thickness of the peat layer was approximately 30–40 cm and the dominating plant species was *Eleocharis interstincta* (approximate coverage of 99%) at both study sites. Nine sampling points (with a distance of 25 m between points) were established at 2,500 m^2 area on both study sites. On the drained site, the three first sampling points were located at 50 m distance from a 2-3 m deep drainage ditch.

Three topsoil cores (0–10 cm layer) from each sampling point were collected and pooled to form a composite sample. In total 18 composite soil samples (approximately 250 g each) were collected. Soil samples were placed into airtight plastic bags. In addition, an intact soil cores were taken for potential dinitrogen (N_2) emission measurements into cylinders (inner Ø 65 mm and height 60 mm) from topsoil (0–10 cm) of each sampling point. All soil samples were kept at cooled conditions (+4°C) during the sampling, storage and transportation by plain (in total 48 hours) to Estonia. In the laboratory of University of Tartu, the samples were divided into subsamples and stored at +4°C (for chemical and N_2 emission analyses) and –20°C (for DNA extraction). Chemical analyses of the soil samples were performed within one week after the sampling. The soil water content (SWC), pH_{KCl} (pH), total carbon (C), Kjeldahl nitrogen (N), ammonium (NH_4-N), nitrate (NO_3-N), total phosphorus (P), total potassium (K), calcium (Ca) and magnesium (Mg) contents were determined in the soil samples using standard procedures[1].

At each sampling point, perforated polyvinyl chloride pipes (Ø 5 cm, sealed in the lower end) as monitoring wells were used to measure the groundwater table depth in the peat layer. Soil temperature was measured at a depth of 10 cm by a temperature logger (Comet Systems Ltd., Rožnov pod Radhoštem, Czech Republic).

Gas emissions measurements

At each soil sampling point, gas samples were collected for nitrous oxide (N_2O) emission measurements twice a day (in the morning and in the afternoon) during three days (totally six sessions) using static closed chambers (Ø 50 cm, height 40 cm and volume 65 L) sealed with a water-filled collar on the soil surface.

The gas samples were taken right after the enclosure of the chambers as well as after 20 min, 40 min and 60 min using pre-evacuated (0.3 mbar) 50-mL gas bottles. The bottles with collected gas samples were transported to Estonia (within 48 h after the last gas sampling session) and analysed in the laboratory of University of Tartu. The targeted gas concentration in the collected air was determined using the Shimadzu GC-2014 gas-chromatographic system (electron capture detector (ECD), flame ionisation detector (FID)) combined with a Loftfield autosampler[2].

Helium atmosphere soil incubation technique[3,4] was used to measure potential N_2 fluxes from soil cores in the same laboratory. The cylinders with the intact soil cores were placed into special gas-tight incubation vessels locating in the climate chamber. Gases were removed by flushing with an artificial gas mixture (21.0% O_2, 358 ppm CO_2, 0.313 ppm N_2O, 1.67 ppm CH_4, 5.97 ppm N_2 and rest He). The new atmosphere equilibrium by continuously flushing the vessel headspace with the artificial gas mixture at 20 mL per min was established after 12–24 h. The flushing time depended on the soil moisture. The temperature was kept similar to the field conditions during the incubation. The gas-chromatograph (Shimadzu GC-2014) equipped with thermal conductivity detector was used to measure N_2 concentration in the mixture of emitted gases accumulated in the headspace (start value, 40 min, 80 min and 120 min as final value) of the cylinder after 2 h of closure. The gas concentration in the chambers increased in a near-linear fashion and linear regression was applied for calculation of the fluxes[4]. The flux measurements with R^2 of 0.81 ($p<0.1$) or greater were used.

Preparation of DNA libraries, sequencing and data processing

A quality check on raw sequence data was performed using FastQC v. 0.11.4[5]. About 1% of paired-end reads had low quality caused by sequencing errors. Quality trimming to remove poly-G tails and reads that have ambiguous nucleotides was done with Cutadapt v. 1.9.1[6]. DNA yield and characteristics of the sequencing data are shown in Supplementary Table 7.

Kaiju v. 1.4.5, which search strategy finds maximum exact matches on the protein-level between query and a reference database (NCBI RefSeq database) using the Borrows-Wheeler transform, was used to classify metagenomic reads down to the species level[7]. 39.8±0.6% (natural site) and 34.3±3.2% (drained site) of the reads were classified with the Kaiju in the heuristic Greedy mode with the default parameter choices, allowing up to five amino acid substitutions during the search.

To screen the metagenomes of the samples for potential to perform nitrogen transformation processes, existing databases of marker genes (amino acid sequences) were used as a reference: *nirK*[8]; *nirS* and *nosZ*[9]; *nifH*, *nrfA*, *hzsA*, bacterial *amoA*/*pmoA* and archaeal *amoA*[10]. Additionally, phylogenetic marker *rpoB* gene (encoding the RNA polymerase) was used as a single copy gene reference[10]. The reference gene datasets alignments were generated using MUSCLE v. 3.8.31[11] and the respective phylogenetic trees were built for detected genes using FastTree v. 2.1.3[12]. Prodigal v. 2.6.2 was used to predict protein-coding regions[13]. Identification of the nitrogen cycling genes from metagenomes was performed with a trained HMM profile for each functional gene using hmmsearch from HMMER v. 3.1b1[14]. Hits were aligned with hmmalign, and placed into reference tree using pplacer v. 1.1.alpha17 with the default parameter choices[15]. 95-100% of the hits (except for drained site *nifH* gene hits (84%)) were unique on average by using dereplicator[16].

ART v. GreatSmokyMountains-04-17-2016 was used to generate simulated sequencing reads dataset based on reference data in order to specify the statistical significance thresholds (E-values of hmmsearch) for limiting false positive matches against reference sequences for each nitrogen cycling

marker gene (0.01 for *nirK*, *nosZ* and archaeal *amoA*; 0.001 for *nirS*, *nifH*, *nrfA* and *hzsA*; 0.0001 for bacterial *amoA*/*pmoA*)[17]. The sensitivity and specificity of the tests were on average 81.6% and 96.7%, respectively. References were tested against different homologs to control their possibility to pick up non-specific sequences and problem occurred in case of *nirK* and *nirS* references, where there is possibility that some of the hits may be homologs and these genera were marked on the phylogenetic trees of *nirK* (Supplementary Fig. 1) and *nirS* (Supplementary Fig. 2).

The overall percentages of the reads matching nitrogen cycle related genes were 28.2±2.6% (natural site) and 29.4±6.0% (drained site). Nitrogen cycling gene read abundances are shown as proportions of total *rpoB* (RNA polymerase) gene read abundance and normalised according to gene length (Figure 4). Abundance-weighted phylogenetic diversity (balance-weighted phylogenetic diversity, BWPD) index values were calculated according to McCoy and Matsen[18] setting Θ=0.5. The variation in the composition of the N-cycling bacterial and archaeal communities among the study sites (beta diversity) was decomposed into replacement, richness difference, and nestedness components[19].

Bacterial and archaeal phyla (profile obtained by metagenomics) abundances according to copy numbers per gram of dry soil of bacterial and archaeal 16S rRNA gene (obtained by qPCR) were shown in Supplementary Fig. 9.

Quantitative PCR

The qPCR assays were performed using RotorGene® Q equipment (Qiagen, Valencia, CA, USA). Stock solutions of target sequence containing plasmids or in case of nifH gene sequence containing synthetic double-stranded DNA fragments (Eurofins MWG Operon, Ebersberg, Germany) were used to create serially diluted standard curves ranging from 25 to 10^9 copies for each target gene (Supplementary Table 9). The qPCR reactions were performed in 10 μL volume containing 5 μL Maxima SYBR Green Master Mix (Thermo Fisher Scientific Inc., Waltham, MA, USA), an optimised concentration of forward and reverse primers (Supplementary Table 8), 1 μL of template DNA, and sterile distilled water. The used gene-specific primer sets, optimised thermal cycling conditions and primer concentrations for each target gene are described in Supplementary Table 8. All qPCR measurements were performed in triplicates for each sample and negative controls were included in every qPCR run.

The quantification data were analysed with RotorGene Series Software v. 2.0.2 (Qiagen) and LinRegPCR program v. 2017.0[20]. For several qPCR assays significant differences in amplification efficiencies of drained and natural peatland site samples were found upon one-way ANOVA analysis ($p<0.05$) (Supplementary Table 9). Consequently, the division of samples into amplicon groups in LinRegPCR analysis based on their origin (natural or drained) was performed to preserve the amplification efficiency estimate for two sample types. Calculation of target gene copy numbers was based on the proposed estimation of fold difference between samples[20] and one-point calibration (OPC) method[21]. The combined calculation protocol was further refined by including multiple

standard points and estimations of amplification efficiencies of both standard dilutions and samples into quantification calculations. In detail, calculation of target gene copy numbers was performed through the estimation of a fold difference (FD) between a sample (A) and multiple data points from the standard curve (B), selected individually for each measured gene. The criteria for selecting standard curve range are following: C_t values of samples must stay at the selected standard range and the smallest standard deviation between the calculated copy numbers of three parallel estimates of each sample defines the most suitable range. Ruijter et al. (2009) proposed a formula which defines FD as:

$FD=N_{0,A}/N_{0,B} = (N_{t,A}/E_A{}^{Ct,A})/(N_{t,B}/E_B C^{t,B})$,

where N_0 marks the starting concentration of the A and B amplicons (in arbitrary fluorescence units), E stands for the amplification efficiencies, N_t stands for the fluorescence threshold values, and C_t marks the fractional number of cycles needed to reach the fluorescence threshold. In order to calculate gene copy numbers in samples, the obtained FD value was multiplied with the known number of gene copies in the respective standard dilution. The final concentration of each target gene was calculated as a mean of all obtained abundance estimates from the range of the standard curve and presented per gram of dry soil (copies/g dw).

The abundance of total prokaryotic organisms was calculated by summing the bacterial and archaeal 16S rRNA gene abundances. The proportion of target genes and microbial groups in soil microbial community was estimated by normalisations against prokaryotic community.

The ratios between two types of *nir* genes (*nirS/nirK*), two *nosZ* gene clades (*nosZI/nosZII*) and *nir* and *nosZ* genes (*nosZ/nir*) were also calculated.

SUPPLEMENTARY TABLES

Supplementary Table 1 Average values (n=9) and standard deviations (in parentheses) of soil chemical and physical parameters in the top 10 cm soil layer of the studied natural and drained site.

Variable	Natural site	Drained site
Soil temperature (°C)	27.1 (0.4)	29.3 (0.1)
Soil water content (%)	77.81 (6.46)	61.03 (4.94)
pH_{KCl}	3.79 (0.08)	3.82 (0.06)
C (%)	35.65 (6.31)	30.79 (3.56)
N (%)	1.61 (0.29)	1.07 (0.18)
C/N	22.24 (1.09)	29.32 (3.97)
NH_4-N (mg/kg)	55.36 (34.27)	8.03 (3.49)
NO_3-N (mg/kg)	0.09 (0.10)	6.40 (8.99)
P (mg/kg)	696 (121)	474 (104)
Ca (mg/kg)	98 (36)	388 (96)
Mg (mg/kg)	65 (31)	451 (211)
K (mg/kg)	154 (38)	132 (54)

Supplementary Table 2 Average values (n=9) and standard deviations (in parentheses) of gene parameter values (obtained by qPCR) in the studied soils. Abbreviation: nd – not detected.

Target gene	Natural site		Drained site	
	Abundance (copies/g dw) or genes ratio	Proportion in the prokaryotic community (%)	Abundance (copies/g dw) or genes ratio	Proportion in the prokaryotic community (%)
Bacterial 16S rRNA	3.46×10^{10} (1.33×10^{10})	43.19 (8.99)	1.12×10^{10} (4.04×10^{09})	10.72 (6.15)
Archaeal 16S rRNA	4.79×10^{10} (2.35×10^{10})	56.81 (8.99)	1.02×10^{11} (2.92×10^{10})	89.28 (6.15)
nirK	9.92×10^{08} (3.17×10^{08})	1.25 (0.19)	6.48×10^{08} (2.12×10^{08})	0.58 (0.14)
nirS	9.38×10^{06} (4.52×10^{06})	0.01143 (0.00342)	4.16×10^{04} (2.06×10^{04})	0.00004 (0.00002)
nosZI	1.28×10^{08} (5.01×10^{07})	0.16 (0.03)	2.60×10^{07} (1.35×10^{07})	0.03 (0.02)
nosZII	3.50×10^{07} (1.13×10^{07})	0.045 (0.012)	7.45×10^{06} (5.93×10^{06})	0.007 (0.006)
nifH	3.79×10^{09} (1.82×10^{09})	4.58 (0.79)	4.23×10^{08} (1.19×10^{08})	0.39 (0.12)
nrfA	3.26×10^{04} (2.40×10^{04})	0.00005 (0.00005)	nd	nd
ANAMMOX 16S rRNA	nd	nd	nd	nd
Bacterial *amoA*	nd	nd	nd	nd
Archaeal *amoA*	1.28×10^{08} (7.25×10^{07})	0.15 (0.07)	2.35×10^{08} (8.63×10^{07})	0.22 (0.09)
Comammox *Nitrospira* clade A *amoA*	nd	nd	nd	nd
Comammox *Nitrospira* clade B *amoA*	nd	nd	nd	nd
nirS/*nirK*	0.00925 (0.00270)	nd	0.00007 (0.00003)	nd
nosZI/*nosZII*	3.69 (1.08)	nd	8.60 (11.08)	nd
nosZ/*nir*	0.16 (0.02)	nd	0.06 (0.03)	nd

Supplementary Table 3 Average values and standard deviations of alpha and beta diversity estimates of nitrogen cycling genes in the natural (N) and drained (D) sites. Abbreviations: BWPD – Balanced weighted phylogenetic diversity index, Repl/BDtotal – total replacement diversity/total beta diversity, RichDiff/BDtotal – total richness difference diversity/total beta diversity, nd – no data.

Diversity estimate	Site	*nirK*	*nirS*	*nosZ*	*nifH*	*nrfA*	Archaeal *amoA*
BWPD (alpha) (n=9)	N	4.03±0.18	1.82±0.17	3.47±0.18	5.07±0.11	1.87±0.13	0.10±0.10
	D	3.28±0.36	2.12±0.22	3.14±0.28	4.35±0.30	nd	0.34±0.07
Repl/BDtotal (%)	All	89.5	66.6	65.6	67.3	nd	61.8
RichDiff/BDtotal (%)	All	10.5	33.4	34.4	32.7	nd	38.2

Supplementary Table 4 Significant relationships (based on Procrustes analyses) between the community structures of the studied functional genes in the tropical peatland soil. The upper triangle of the table contains the Procrustes sum of squares (M^2) and the lower triangle contains the corresponding p-values.

	nirK	*nirS*	*nosZ*	*nifH*	Archaeal *amoA*
nirK		0.53	0.31	0.41	0.43
nirS	0.0004		0.54	0.36	0.55
nosZ	0.0001	0.0007		0.38	0.47
nifH	0.0002	0.0001	0.0001		0.42
Archaeal *amoA*	0.0004	0.0012	0.0003	0.0002	

Supplementary Table 5 Statistically significant Spearman's rank correlations (n=9) between target gene abundances (copies/g dw) (obtained by qPCR) and environmental factors in the natural and drained sites. Abbreviations: N_2 – potential N_2 emission rate.

Site	Physicochemical variables or target gene abundance or gaseous parameter	Target gene abundance							
		16S bacteria	16S archaea	*nirS*	*nirK*	*nosZI*	*nosZII*	*nifH*	*amoA* archaea
Natural	pH	-0.85**		-0.85**		-0.93***		-0.80**	-0.79*
	C	0.77*	0.67*	0.85**		0.92***		0.83**	0.77*
	N	0.68*		0.78*		0.87**		0.78*	0.68*
	NH_4-N	0.72*		0.70*		0.80**		0.83**	
	NO_3-N							-0.72*	
	Mg			0.75*		0.77*			0.73*
	K	0.70*		0.77*		0.83**		0.72*	0.82**
	nirS		0.83**		0.75*	0.97***	0.67*	0.70*	0.85**
	nirK		0.98***	0.75*		0.75*	0.88**	0.80**	0.75*
	nosZI	0.75*	0.82**	0.97***	0.75*			0.82**	0.87**
	nosZII		0.85**	0.67*	0.88**				
	nifH	0.92***	0.77*	0.70*	0.80**	0.82**			0.67*
	amoA archaea	0.67*	0.83**	0.85**	0.75*	0.87**		0.67*	
	N_2			0.71*		0.70*			
Drained	Soil temp							0.79*	0.84**
	P						-0.72*		
	nosZI		-0.77*						
	amoA archaea							0.80**	
	N_2						0.86**		

* – $p<0.05$; ** – $p<0.01$; *** – $p<0.001$

Supplementary Table 6 Statistically significant Spearman's rank correlations (n=9) between target gene proportions and ratios (obtained by qPCR), and environmental factors in the natural and drained sites. Abbreviations: SWC – soil water content, N_2O – N_2O emission rate, N_2 – potential N_2 emission rate.

Site	Physicochemical variables or target gene proportion or gaseous parameter	Target gene proportion								Target genes ratio		
		16S bacteria	16S archaea	*nirS*	*nirK*	*nosZII*	*nifH*	*nrfA*	*amoA* archaea	*nirS*/*nirK*	*nosZI*/*nosZII*	*nosZ*/*nir*
Natural	Soil temperature					-0.69*						
	pH				0.82**						-0.77*	-0.77*
	C											0.80**
	N										0.67*	0.85**
	NH_4-N							-0.80**				0.75*
	NO_3-N					0.79*		0.74*				
	P							-0.68*	0.78*			
	Mg									0.67*		
	K							-0.68*				0.82**
	nirK										-0.80**	
	N_2O			0.68*				0.77*				
	N_2									0.80**		0.74*
Drained	C				0.82**							
	N	-0.71*	0.71*				-0.69*		-0.79*			-0.73*
	C/N	0.67*	-0.67*			0.77*	0.73*					
	NO_3-N	-0.68*	0.68*				-0.67*		-0.75*			-0.75*
	P					-0.82**					0.77*	
	16S bacteria											0.72*
	16S archaea											-0.72*
	nifH											0.75*
	amoA archaea											0.85**
	N_2					0.92***					-0.73*	

* – p<0.05; ** – p<0.01; *** – p<0.001

Supplementary Table 7 Average values and standard deviations of DNA yield and characteristics of the sequencing (NextSeq 500 Illumina) data (total number of original and quality filtered reads, average length of quality filtered reads) for studied samples of natural (N) and drained (D) peatland sites.

Sample	DNA conc. (ng/ μL) (n=3)	Number of reads	After quality trimming	
			Number of reads	Average reads length (bp)
N.1.1	12.43±2.38	9506177	9046123	150.27
N.1.2	18.47±6.26	14953635	14341172	150.24
N.1.3	14.40±3.02	10572853	10149118	150.39
N.2.1	18.10±3.83	11151239	10731841	150.25
N.2.2	11.47±1.53	14144758	13365036	150.08
N.2.3	14.67±4.97	12548896	11713611	150.34
N.3.1	17.83±2.14	11478728	10944296	150.25
N.3.2	12.87±1.53	12359785	11517305	150.24
N.3.3	16.03±4.57	11389397	10848463	150.12
D.1.1	11.93±2.66	12349474	11759191	150.41
D.1.2	11.40±2.40	10252354	9474796	150.41
D.1.3	10.43±1.12	11726055	11019556	150.41
D.2.1	14.90±1.37	11701504	11168947	150.46
D.2.2	9.87±3.09	10295396	9638683	150.38
D.2.3	10.60±3.46	13616686	12804467	150.38
D.3.1	10.10±2.78	11630656	11021442	150.44
D.3.2	11.07±2.63	11895173	11215670	150.38
D.3.3	11.17±3.68	13095542	12453931	150.48

Supplementary Table 8 Characteristics of the used qPCR primers and programs.

Target gene	Primer	Amplicon size (bp)	Primer conc. (μM)	qPCR program
Bacterial 16S rRNA	Bact517F[22]<br>Bact1028R[23]	530	0.6	95°C 10 min; 35 cycles: 95°C 30 s; 60°C 45 s; 72°C 45s
Archaeal 16S rRNA	Arc519F[24]<br>Arch910R[24]	393	0.6	95°C 10 min; 45 cycles: 95°C 15 s; 56°C 30 s; 72°C 30s
nirS	nirSC1F[25]<br>nirSR3cd[26]	431	0.8	95°C 10 min; 45 cycles: 95°C 15 s; 58°C 30 s; 72°C 30s, 80°C 30 s[a]
nirK	nirK876[27]<br>nirK1040[27]	165	0.8	95°C 10 min; 45 cycles: 95°C 15 s, 58°C 30 s, 72°C 30 s, 80°C 30 s[a]
nosZI	nosZ2F[28]<br>nosZ2R[28]	267	0.8	95°C 10 min; 45 cycles: 95°C 15 s, 60°C 30 s, 72°C 30 s, 80°C 30 s[a]
nosZII	nosZIIF[29]<br>nosZIIR[29]	~700	0.8	95°C 10 min; 45 cycles: 95°C 30 s, 54°C 45 s, 72°C 45 s, 80°C 45 s[a]
nifH	Ueda19F[30]<br>Ueda407R[30]	390	0.8	95°C 10 min; 45 cycles: 95°C 30 s, 53°C 45 s, 72°C 45 s
nrfA	6F[31]<br>6R[31]	222	0.8	95°C 10 min; 45 cycles: 95°C 15 s, 55°C 30 s, 72°C 30 s
ANAMMOX 16S rRNA	A438F[32]<br>A684R[32]	248	0.9	95°C 10 min; 45 cycles: 95°C 15 s, 51°C 30 s, 72°C 30 s
Bacterial *amoA*	amoA-1F[33]<br>amoA-2R[33]	491	0.8	95°C 10 min; 45 cycles: 95°C 30 s, 60°C 45 s, 72°C 45 s
Archaeal *amoA*	CrenamoA 23F[34]<br>CrenamoA 616R[34]	~600	0.8	95°C 10 min; 45 cycles: 95°C 30 s, 55°C 45 s, 72°C 45 s
Comammox *Nitrospira* clade A *amoA*	comaA-244F[35]<br>comaA-659R[35]	415	0.5–0.9[b]	95°C 10 min; 45 cycles: 95°C 30 s, 46-52°C[b] 45 s, 72°C 60 s
Comammox *Nitrospira* clade B *amoA*	comaB-244F[35]<br>comaB-659R[35]	415	0.5–0.9[b]	95°C 10 min; 45 cycles: 95°C 30 s, 50-52°C[b] 45 s, 72°C 60 s

[a] Fluorescence signal was read after the second extension step (80 °C); [b] Range of tested primer concentrations and annealing temperatures

Supplementary Table 9 The range of the standard curves used for target gene copy numbers calculations as well as means and standard deviations of qPCR amplification efficiencies (according to LinRegPCR program v.2017.0) for the standard dilutions and studied samples of natural and drained peatland sites. Abbreviation: nd – not detected.

Target gene	Standard curve range	qPCR efficiency		
		Standard dilutions	Natural site (n=27)	Drained site (n=27)
Bacterial 16S rRNA	10^4–10^8	1.866±0.031 (n=12)	1.835±0.031	1.852±0.023
Archaeal 16S rRNA	10^7–10^9	1.754±0.020 (n=7)	1.817±0.054	1.807±0.037
nirK	10^5–10^7	1.663±0.046 (n=9)	1.666±0.045	1.713±0.054
nirS	10^2–10^4	1.738±0.038 (n=6)	1.776±0.044	1.822±0.072 (n=21)
nosZI	10^3–10^5	1.686±0.038 (n=9)	1.680±0.039	1.689±0.045
nosZII	10^5–10^8	1.611±0.040 (n=11)	1.622±0.050 (n=25)	1.611±0.050 (n=22)
nifH	10^6–10^8	1.666±0.030 (n=9)	1.726±0.036	1.747±0.035 (n=26)
nrfA	25–10^2	1.771±0.030 (n=6)	1.801±0.100 (n=20)	nd
Archaeal *amoA*	10^4–10^6	1.815±0.034 (n=9)	1.812±0.063	1.816±0.040

SUPPLEMENTARY FIGURES

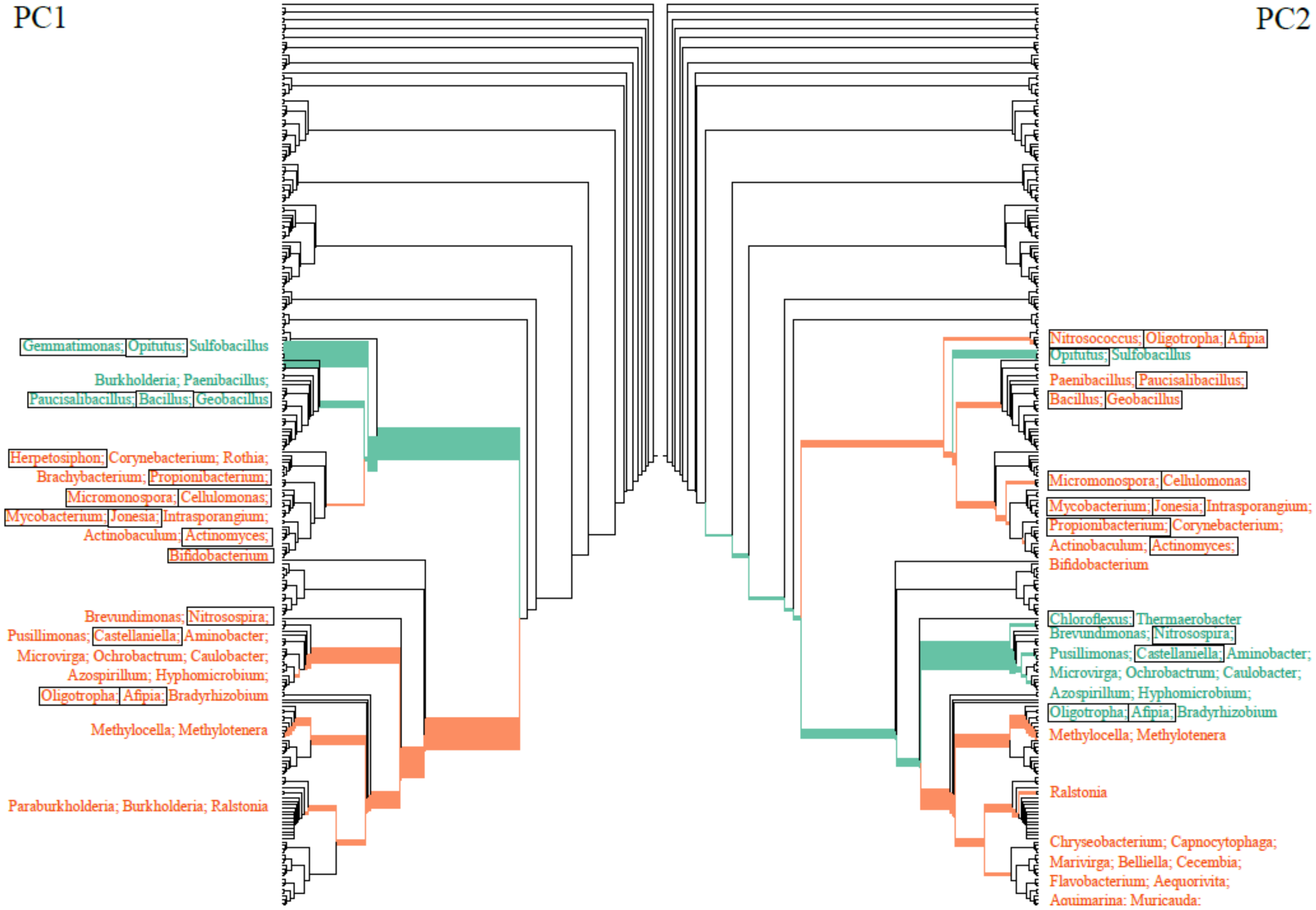

Supplementary Fig. 1 The phylogenetic tree of *nirK* sequences classified at genus level. Orange colour marks genera that were in positive direction of the principal component axes 1 and 2 of PCA plot on Fig. 5 and green colour marks genera that were in negative direction of the principal component axes 1 and 2 of PCA plot on Fig. 5, and thickened proportional to magnitude. Genera in little black boxes are genera that may include some amounts of homologous sequence data.

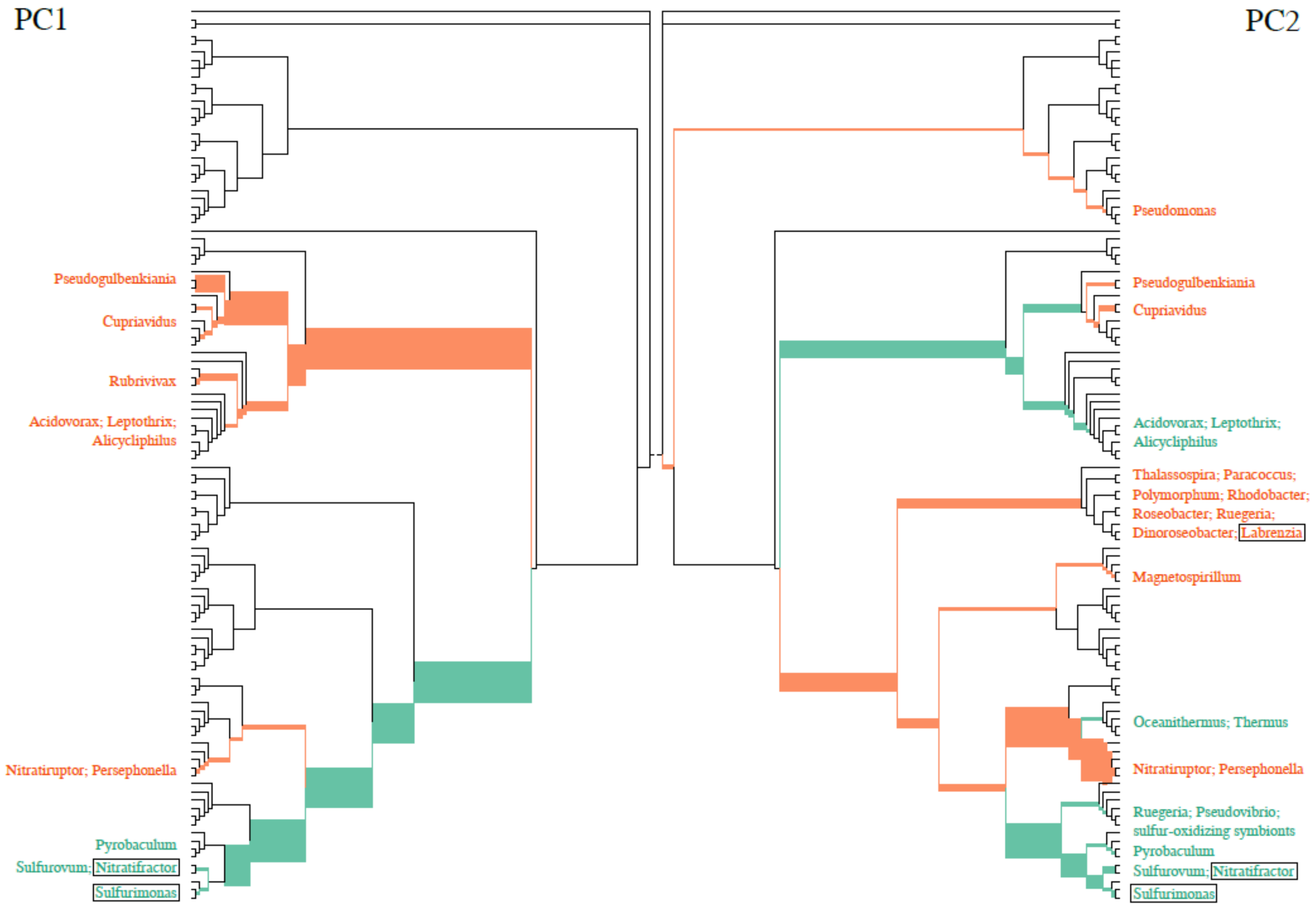

Supplementary Fig. 2 The phylogenetic tree of *nirS* sequences classified at genus level. Orange colour marks genera that were in positive direction of the principal component axes 1 and 2 of PCA plot on Fig. 5 and green colour marks genera that were in negative direction of the principal component axes 1 and 2 of PCA plot on Fig. 5, and thickened proportional to magnitude. Genera in little black boxes are genera that may include some amounts of homologous sequence data.

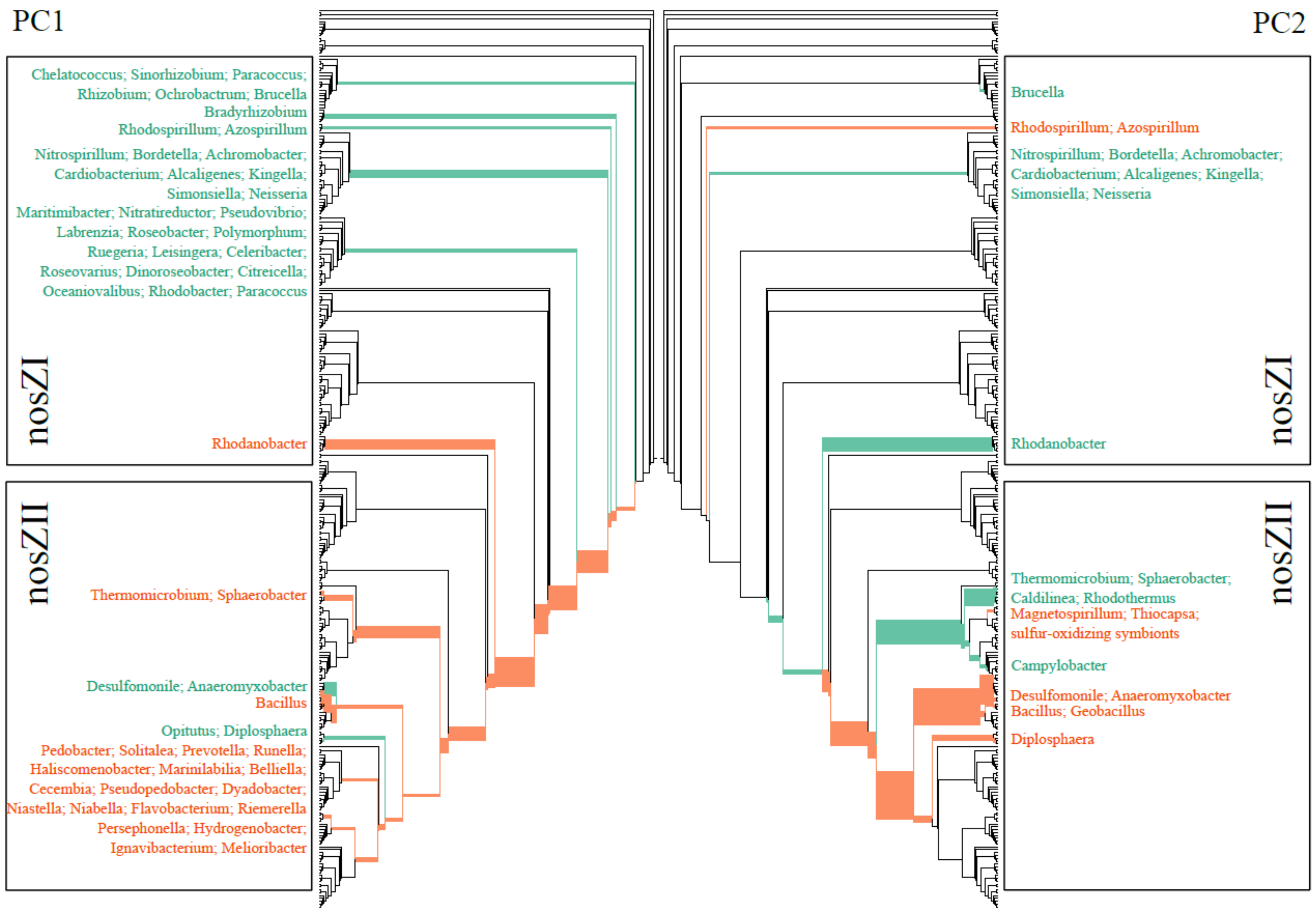

Supplementary Fig. 3 The phylogenetic tree of *nosZI* and *nosZII* sequences classified at genus level. Orange colour marks genera that were in positive direction of the principal component axes 1 and 2 of PCA plot on Fig. 5 and green colour marks genera that were in negative direction of the principal component axes 1 and 2 of PCA plot on Fig. 5, and thickened proportional to magnitude.

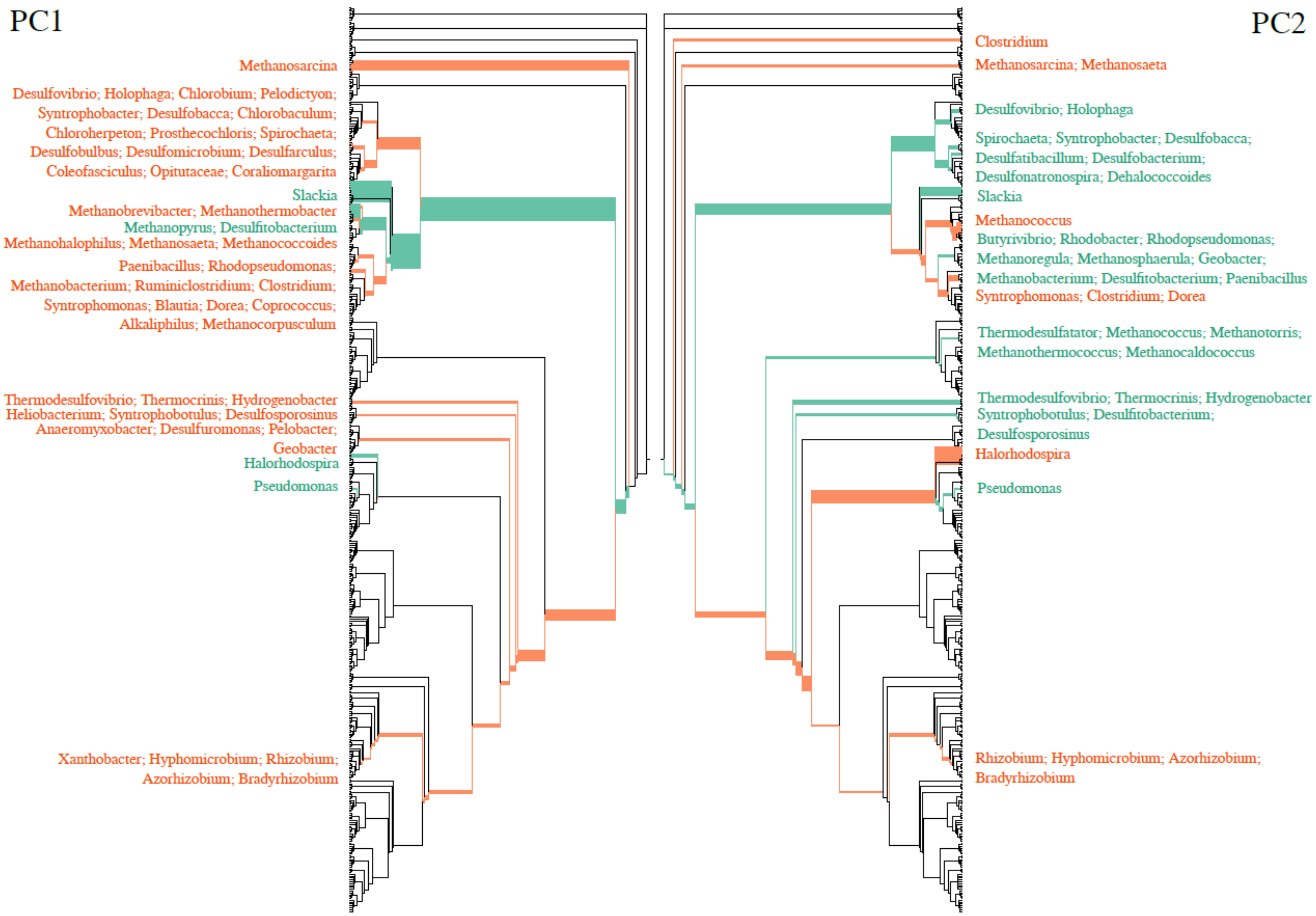

Supplementary Fig. 4 The phylogenetic tree of *nifH* sequences classified at genus level. Orange colour marks genera that were in positive direction of the principal component axes 1 and 2 of PCA plot on Fig. 5 and green colour marks genera that were in negative direction of the principal component axes 1 and 2 of PCA plot on Fig. 5, and thickened proportional to magnitude.

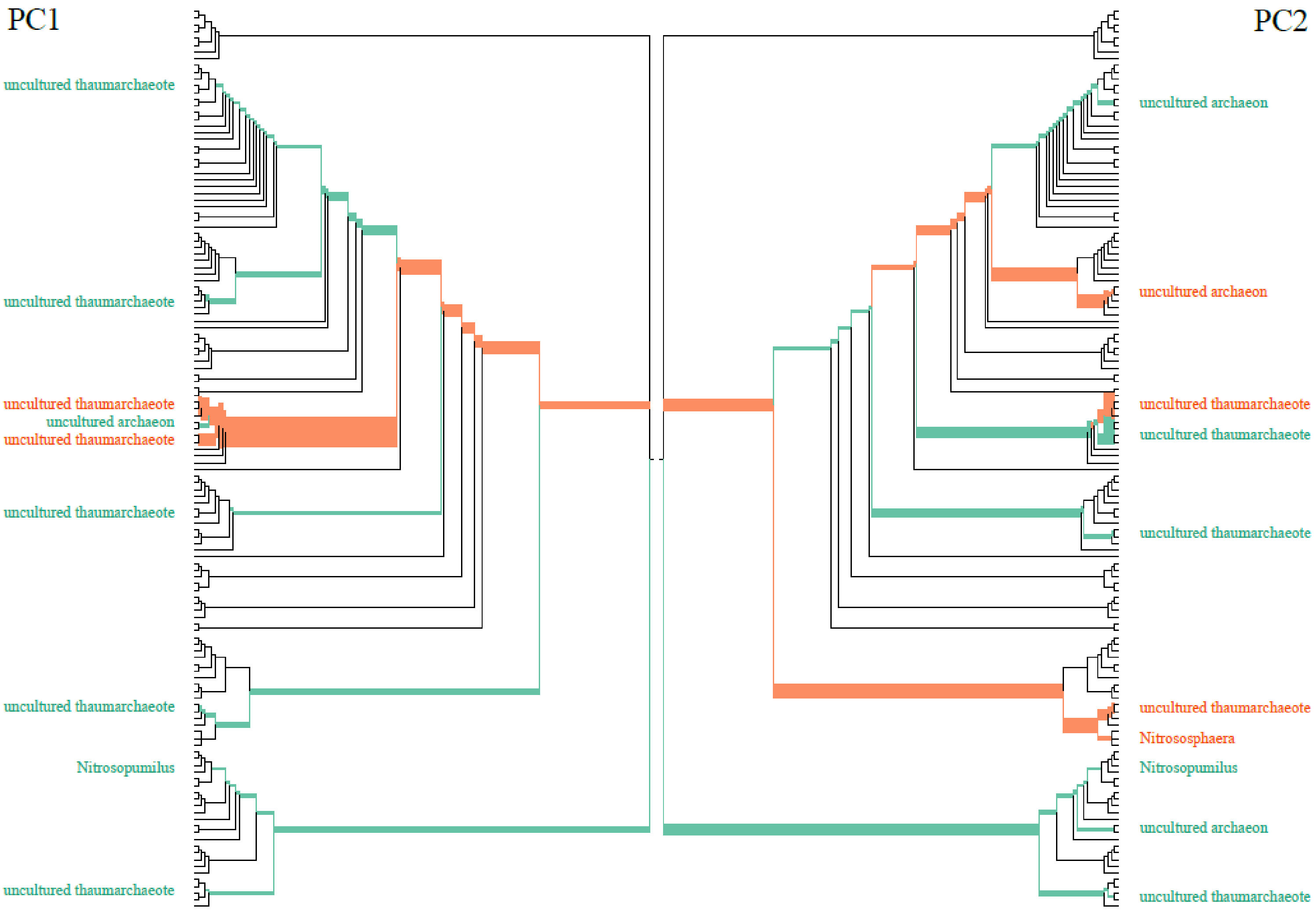

Supplementary Fig. 5 The phylogenetic tree of *amoA* sequences classified at genus level. Orange colour marks genera that were in positive direction of the principal component axes 1 and 2 of PCA plot on Fig. 5 and green colour marks genera that were in negative direction of the principal component axes 1 and 2 of PCA plot on Fig. 5, and thickened proportional to magnitude.

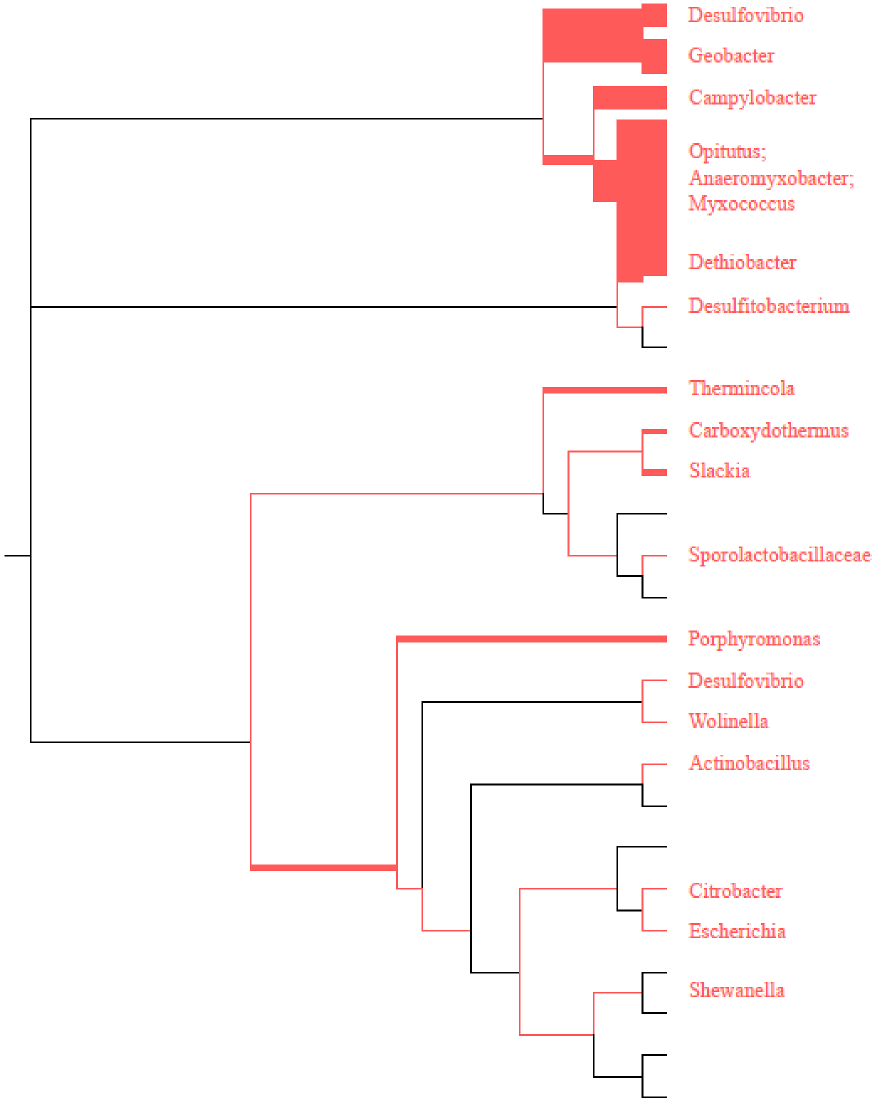

Supplementary Fig. 6 The phylogenetic tree of *nrfA* sequences classified at genus level. Genera detected from natural site are coloured and thickened proportional to magnitude.

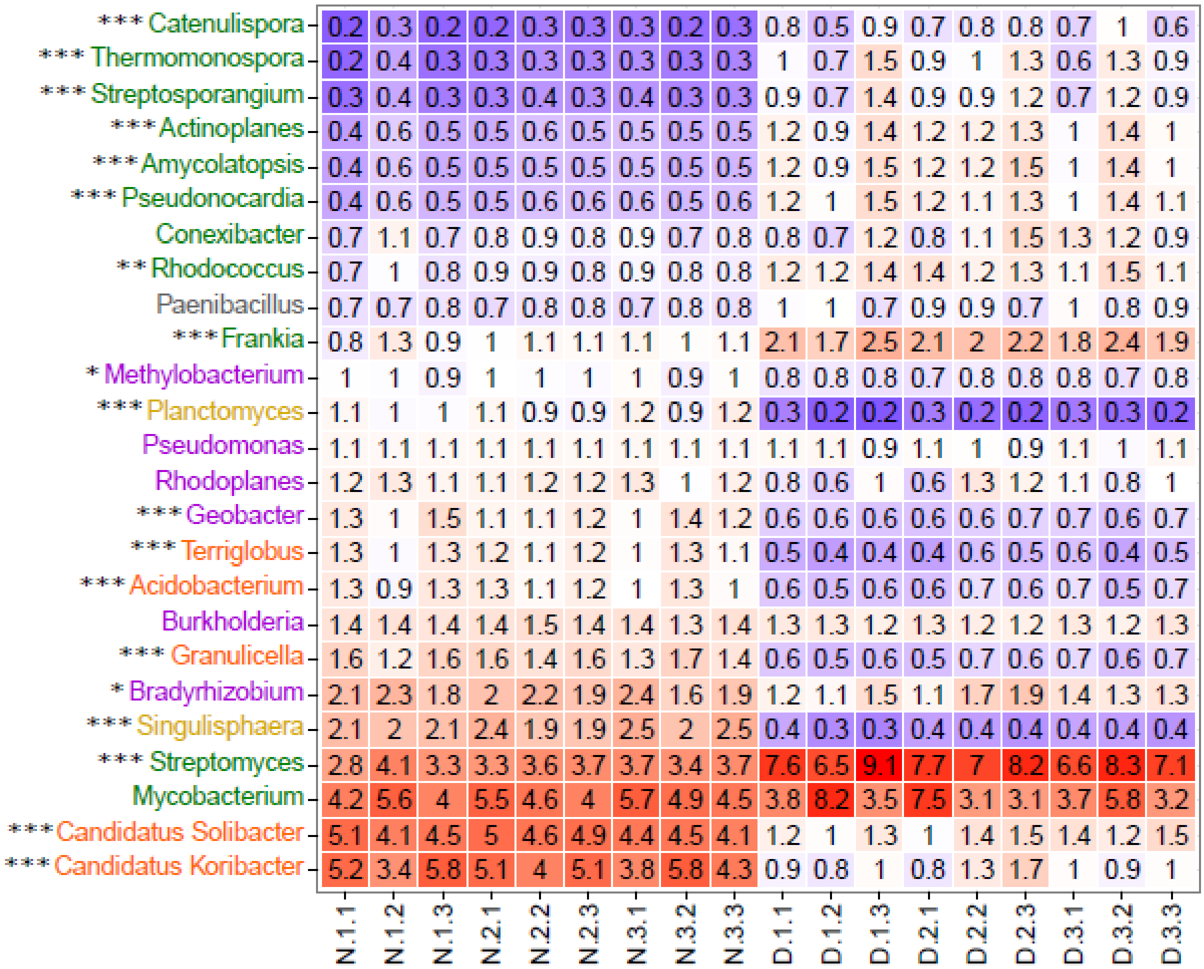

Supplementary Fig. 7 Distribution of dominant bacterial genera among samples of natural (N) and drained sites (D). The relative sequence abundances (≥1% at least in one sample) are visualised using a blue-white-red colour scale. Bacterial genera are coloured by their respective phylum (green – *Actinobacteria*, orange – *Acidobacteria*, yellow – *Planctomycetes*, purple – *Proteobacteria*, grey – *Firmicutes*). Asterisks show statistically significant difference (* – p<0.05; ** – p<0.01; *** – p<0.001) between the natural and drained site.

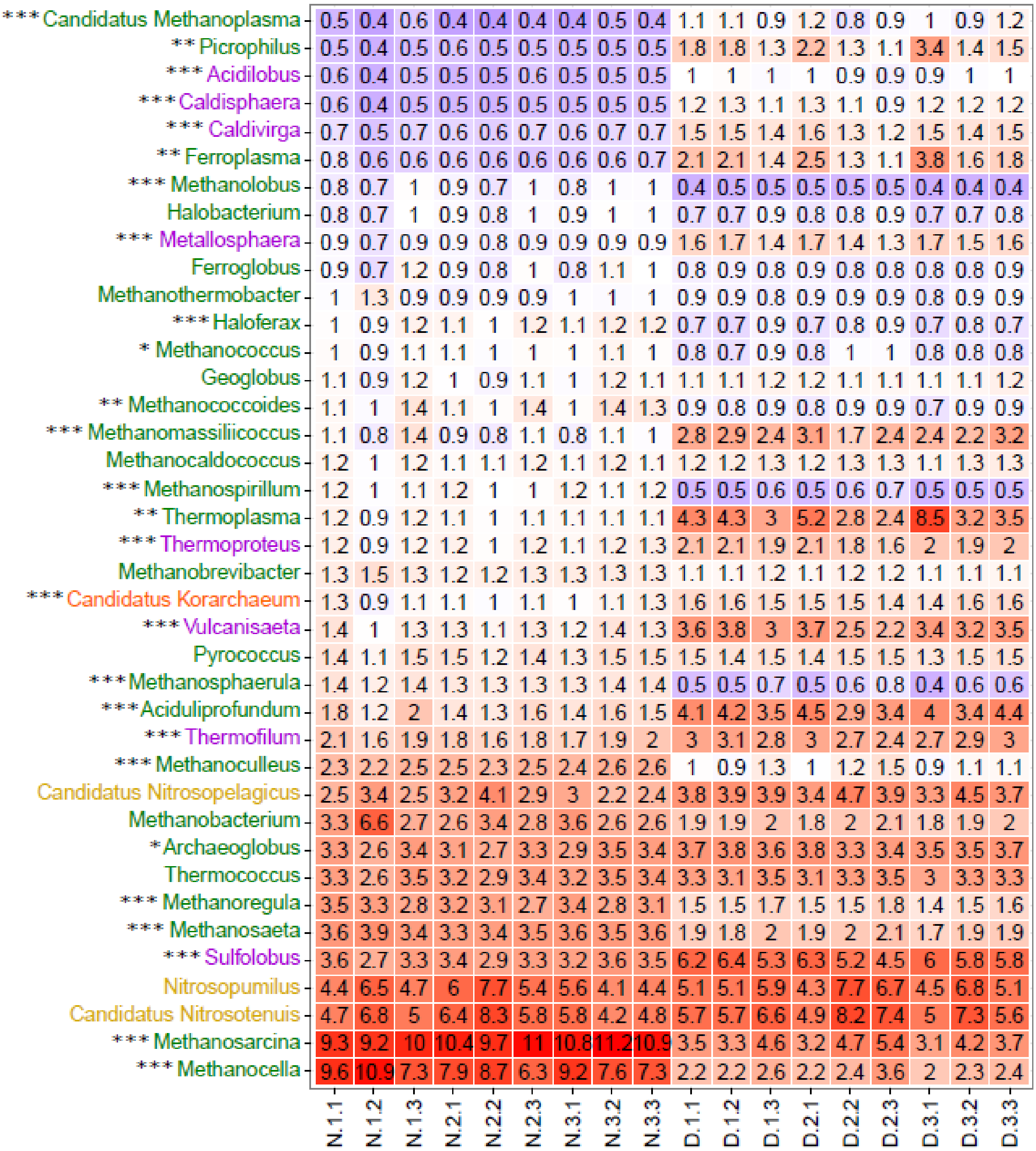

Supplementary Fig. 8 Distribution of dominant archaeal genera among samples of natural (N) and drained sites (D). The relative sequence abundances (≥1% at least in one sample) are visualised using a blue-white-red colour scale. Bacterial genera are coloured by their respective phylum green – *Euryarchaeota*, yellow – *Thaumarchaeota*, orange – *Korarchaeota*, purple – *Crenarchaeota*). Asterisks show statistically significant difference (* – $p<0.05$; ** – $p<0.01$; *** – $p<0.001$) between the natural and drained site.

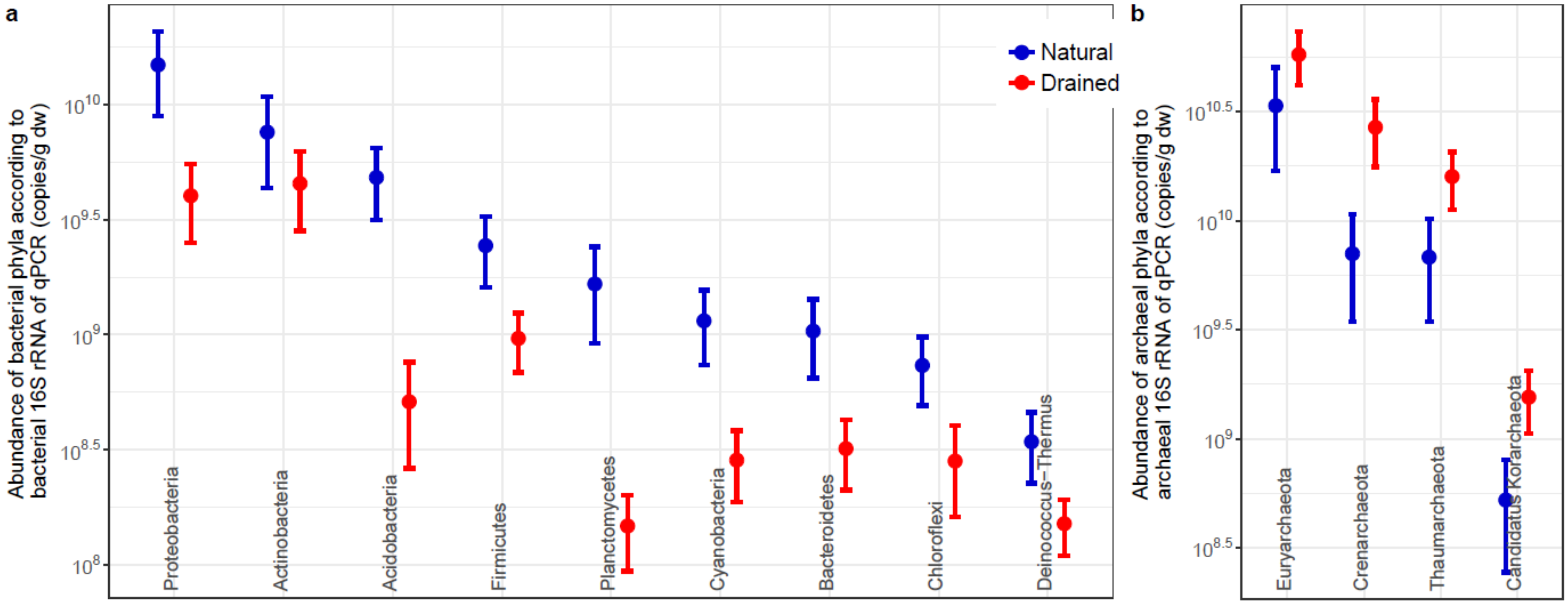

Supplementary Fig. 9 Average values (n=9) and standard deviations of bacterial (a) and archaeal (b) phyla abundances according to copy numbers per gram of dry soil of bacterial and archaeal 16S rRNA gene in the studied soils. Calculated values are based on phylum relative abundance obtained by metagenomics and 16S rRNA gene copy numbers obtained by qPCR.

REFERENCES

1. APHA. *Standard Methods for the Examination of Water and Waste Water*. (American Public Health Organisation, American Water Works Assocation, Water Environment Federation, 1989).
2. Loftfield, N., Flessa, H., Augustin, J. & Beese, F. Automated gas chromatographic system for rapid analysis of the atmospheric trace gases methane, carbon dioxide, and nitrous oxide. *J. Environ. Qual.* **26,** 560–564 (1997).
3. Swerts, M., Uytterhoeven, G., Merckx, R. & Vlassak, K. Semicontinuous measurement of soil atmosphere gases with gas-flow soil core method. *Soil Sci. Soc. Am. J.* **59,** 1336–1342 (1995).
4. Butterbach-Bahl, K., Willibald, G. & Papen, H. Soil core method for direct simultaneous determination of N2 and N2O-emissions from forest soil. *Plant Soil* **240,** 105–116 (2002).
5. Andrews, S. FastQC: a quality control tool for high throughput sequence data. (2010).
6. Martin, M. Cutadapt removes adapter sequences from high-throughput sequencing reads. *EMBnet.journal* **17,** 10 (2011).
7. Menzel, P., Ng, K. L. & Krogh, A. Kaiju: Fast and sensitive taxonomic classification for metagenomics. *bioRxiv* **7,** 1–9 (2015).
8. Decleyre, H., Heylen, K., Tytgat, B. & Willems, A. Highly diverse nirK genes comprise two major clades that harbour ammonium-producing denitrifiers. *BMC Genomics* **17,** 155 (2016).
9. Graf, D. R. H., Jones, C. M. & Hallin, S. Intergenomic comparisons highlight modularity of the denitrification pathway and underpin the importance of community structure for N_2O emissions. *PLoS One* **9,** e114118 (2014).
10. Lüke, C., Speth, D. R., Kox, M. A. R., Villanueva, L. & Jetten, M. S. M. Metagenomic analysis of nitrogen and methane cycling in the Arabian Sea oxygen minimum zone. *PeerJ* **4,** e1924 (2016).
11. Edgar, R. C. MUSCLE: Multiple sequence alignment with high accuracy and high throughput. *Nucleic Acids Res.* **32,** 1792–1797 (2004).
12. Price, M. N., Dehal, P. S. & Arkin, A. P. FastTree 2 - Approximately maximum-likelihood trees for large alignments. *PLoS One* **5,** e9490 (2010).
13. Hyatt, D. *et al.* Prodigal: prokaryotic gene recognition and translation initiation site

identification. *BMC Bioinformatics* **11,** 119 (2010).

14. Eddy, S. R. Accelerated profile HMM searches. *PLoS Comput. Biol.* **7,** e1002195 (2011).
15. Matsen IV, F. A. & Evans, S. N. Edge Principal Components and Squash Clustering: Using the Special Structure of Phylogenetic Placement Data for Sample Comparison. *PLoS One* **8,** e56859 (2013).
16. Fish, J. A. *et al.* FunGene : the functional gene pipeline and repository. *Front. Microbiol.* **4,** 1–14 (2013).
17. Huang, W., Li, L., Myers, J. R. & Marth, G. T. ART: A next-generation sequencing read simulator. *Bioinformatics* **28,** 593–594 (2012).
18. Mccoy, C. O. & Matsen IV, F. A. Abundance-weighted phylogenetic diversity measures distinguish microbial community states and are robust to sampling depth. *PeerJ* **1,** e157 (2013).
19. Legendre, P. & Gauthier, O. Statistical methods for temporal and space – time analysis of community composition data. *Proc. R. Soc. London B Biol. Sci.* **281,** 20132728 (2014).
20. Ruijter, J. M. *et al.* Amplification efficiency: Linking baseline and bias in the analysis of quantitative PCR data. *Nucleic Acids Res.* **37,** e45 (2009).
21. Brankatschk, R., Bodenhausen, N., Zeyer, J. & Bürgmann, H. Simple Absolute Quantification Method Correcting for Quantitative PCR Efficiency Variations for Microbial Community Samples. *Appl. Environ. Microbiol.* **78,** 4481–4489 (2012).
22. Liu, Z., Lozupone, C., Hamady, M., Bushman, F. D. & Knight, R. Short pyrosequencing reads suffice for accurate microbial community analysis. *Nucleic Acids Res.* **35,** e120 (2007).
23. Dethlefsen, L., Huse, S., Sogin, M. L. & Relman, D. A. The Pervasive Effects of an Antibiotic on the Human Gut Microbiota, as Revealed by Deep 16S rRNA Sequencing. *PLoS Biol.* **6,** e280 (2008).
24. Espenberg, M. *et al.* Impact of Reed Canary Grass Cultivation and Mineral Fertilisation on the Microbial Abundance and Genetic Potential for Methane Production in Residual Peat of an Abandoned Peat Extraction Area. *PLoS One* **11,** e0163864 (2016).
25. Wei, W. *et al.* Higher diversity and abundance of denitrifying microorganisms in environments than considered previously. *ISME J.* **9,** 1954–1965 (2015).
26. Kandeler, E., Deiglmayr, K., Tscherko, D., Bru, D. & Philippot, L. Abundance of narG, nirS, nirK, and nosZ genes of denitrifying bacteria during primary successions of a

glacier foreland. *Appl. Environ. Microbiol.* **72,** 5957–5962 (2006).

27. Henry, S. *et al.* Quantification of denitrifying bacteria in soils by nirK gene targeted real-time PCR. *J. Microbiol. Methods* **59,** 327–335 (2004).
28. Henry, S., Bru, D., Stres, B., Hallet, S. & Philippot, L. Quantitative detection of the nosZ gene, encoding nitrous oxide reductase, and comparison of the abundances of 16S rRNA, narG, nirK, and nosZ genes in soils. *Appl. Environ. Microbiol.* **72,** 5181–5189 (2006).
29. Jones, C. M., Graf, D. R. H., Bru, D., Philippot, L. & Hallin, S. The unaccounted yet abundant nitrous oxide-reducing microbial community: a potential nitrous oxide sink. *ISME J.* **7,** 417–426 (2013).
30. Ueda, T., Suga, Y., Yahiro, N. & Matsuguchi, T. Remarkable N_2-fixing bacterial diversity detected in rice roots by molecular evolutionary analysis of *nifH* gene sequences. *J. Bacteriol.* **177,** 1414–1417 (1995).
31. Takeuchi, J. Habitat Segregation of a Functional Gene Encoding Nitrate Ammonification in Estuarine Sediments. *Geomicrobiol. J.* **23,** 75–87 (2006).
32. Humbert, S., Zopfi, J. & Tarnawski, S. E. Abundance of anammox bacteria in different wetland soils. *Environ. Microbiol. Rep.* **4,** 484–490 (2012).
33. Rotthauwe, J., Witzel, K. & Liesack, W. The Ammonia Monooxygenase Structural Gene amoA as a Functional Marker: Molecular Fine-Scale Analysis of Natural Ammonia-Oxidizing Populations. **63,** 4704–4712 (1997).
34. Tourna, M., Freitag, T. E., Nicol, G. W. & Prosser, J. I. Growth, activity and temperature responses of ammonia-oxidizing archaea and bacteria in soil microcosms. *Environ. Microbiol.* **10,** 1357–1364 (2008).
35. Pjevac, P. *et al.* *AmoA*-targeted polymerase chain reaction primers for the specific detection and quantification of comammox *Nitrospira* in the environment. *bioRxiv* 96891 (2017). doi:10.1101/096891
